# Association of leisure time physical activity with gut microbiota composition in early adulthood

**DOI:** 10.1038/s41598-025-02287-2

**Published:** 2025-06-04

**Authors:** Hanna-Mari Boelius, Anna-Katariina Aatsinki, Marja A. Heiskanen, Eero A. Haapala, Eveliina Munukka, Juha Mykkänen, Noora Kartiosuo, Leo Lahti, Anniina Keskitalo, Pentti Huovinen, Harri Niinikoski, Jorma Viikari, Tapani Rönnemaa, Hanna Lagström, Antti Jula, Suvi P. Rovio, Olli T. Raitakari, Katja Pahkala

**Affiliations:** 1 Centre for Population Health Research, University of Turku and Turku University Hospital, Turku, Finland; 2 Research Centre of Applied and Preventive Cardiovascular Medicine, University of Turku, Turku, Finland; 3 FinnBrain Birth Cohort Study, Turku Brain and Mind Center, Department of Clinical Medicine, University of Turku and Turku University Hospital, Turku, Finland; 4 Faculty of Sport and Health Sciences, University of Jyväskylä, Jyväskylä, Finland; 5 Institute of Biomedicine, School of Medicine, University of Eastern Finland, Kuopio, Finland; 6 Microbiome Biobank, Institute of Biomedicine, University of Turku, Turku, Finland; 7 Department of Clinical Microbiology, Turku University Hospital, Turku, Finland; 8 Department of Mathematics and Statistics, University of Turku, Turku, Finland; 9 Department of Computing, Faculty of Technology, University of Turku, Turku, Finland; 10 Department of Pediatrics and Adolescent Medicine, Turku University Hospital, University of Turku, Turku, Finland; 11 Department of Medicine, University of Turku, Turku, Finland; 12 Division of Medicine, Turku University Hospital, Turku, Finland; 13 Department of Public Health, University of Turku, Turku University Hospital, Turku, Finland; 14 Nutrition and Food Research Center, Faculty of Medicine, University of Turku, Turku, Finland; 15 Department of Chronic Disease Prevention, Institute for Health and Welfare, Turku, Finland; 16 Department of Clinical Physiology and Nuclear Medicine, Turku University Hospital, Turku, Finland; 17 Paavo Nurmi Centre and Unit for Health and Physical Activity, University of Turku, Turku, Finland

**Keywords:** Gut microbiota, Physical activity, Diet, Cohort study, Health, Microbiota, Health care

## Abstract

Physical activity is associated with compositional changes in gut microbiota. Studies applying data from general populations exhibiting varying physical activity levels are scarce. This study investigated the associations between leisure time physical activity and gut microbiota composition in a general population of young adults. Faecal samples were analyzed with 16 S rRNA sequencing and leisure time physical activity were assessed in *N* = 302 (176 females, 126 males) 26-year-old individuals of European descent. Leisure time physical activity was assessed using a self-administrated questionnaire and expressed as metabolic equivalent (MET) h/wk. Participants were defined as inactive (*N* = 83) and active (*N* = 219) based on estimation to meet the physical activity guidelines. Diet was assessed using food diaries. First, associations of physical activity and gut microbiota were investigated and then the models were further adjusted for diet (*N* = 280). While gut microbiota diversity was similar, community composition differed between the active and inactive participants. The association diluted after adjustment for diet. Moreover, 16 genera, e.g., *Lactobacillus*,* Veillonella*, and *Prevotella* were more abundant in the active compared with the inactive participants. After adjustment for diet, these associations, except *Barnesiella*, were no longer detected, but other genera were found. In conclusion, physical activity in young adults is associated with gut microbiota community composition and an increase in the abundance of genera considered beneficial for overall health. The associations are partly confounded by diet.

**Supplementary Information:**

The online version contains supplementary material available at 10.1038/s41598-025-02287-2.

## Introduction

Gut microbiota (GM) functions in nutrient and energy utilisation and uptake, vitamin synthesis, inflammatory modulation as well as in host immune response^1^. Moreover, the composition of an individual’s GM profile is influenced by a wide range of factors, including dietary habits, antibiotic use, body mass index (BMI), and lifestyle factors, such as physical activity (PA)^2^.

PA is an effective means to promote health and lower the risk of many chronic conditions, such as cardiovascular, metabolic, and mental diseases^3^. Interestingly, GM may be one of the transmitters of the overall health benefits that PA inflicts^4^. For example, GM composition in athletes is richer with potentially health-promoting species and higher in diversity compared with non-athletes^5^. There are several hypothesised pathways for how PA could alter GM. These pathways include weight loss, maintenance of glucose homeostasis, excretion of bile acid and short-chain fatty acids (SCFA), secretion of myokines, activation of muscle toll-like receptors by lipopolysaccharides, increased immunoglobulin A production, activation of the hypothalamic–pituitary–adrenal axis, heat stress and gut transit time reduction, as previously reviewed^6^. It has also been suggested that GM may have a beneficial effect on physical performance through processes such as the availability of SCFAs, the amount of muscle glycogen, the activity of antioxidant enzymes, gastrointestinal permeability, and lactate metabolism^7^. Above mentioned pathways between PA and GM, such as the availability of SCFA and excretion of bile acids, are likely confounded by diet^6^. Collectively, it is well known that diet relates to GM^8^ and it has been recently shown that those with greater compliance to Dietary Guidelines for Americans demonstrated higher diversity in their GM and a greater abundance of bacteria capable of metabolising complex carbohydrates^9^.

Collectively, there is growing evidence indicating a relationship between PA and GM^4,10^. However, most previous studies have been conducted on elderly or overweight and obese individuals, and among athletes, whereas only a few studies have focused on a general population of young adults^5,10-13^. Despite the growing evidence that links vigorous or long-lasting PA and GM, further insight is needed on e.g., how a moderate amount of PA is associated with GM composition among individuals representing the general population. Hence, this study aims to explore if leisure time PA (LTPA) associates with GM composition in a population-based cohort of young adults. Specifically, we investigate whether having a PA level consistent with the minimum recommended amount or higher is associated with differences in GM composition compared to having a low PA level. We expect to find similar associations as in other populations but potentially with more moderate effect sizes. Finally, we test if taking diet into account modifies the observed associations between PA and GM since a healthy diet and physical activity tend to cluster. We anticipate that the associations are confounded by diet to a certain extent.

## Methods

### Study sample

This study utilises data from the ongoing randomised Special Turku Coronary Risk Factor Intervention Project (STRIP), which investigates the long-term effects of infancy-onset dietary counseling on cardiometabolic health^14^. In brief, families of five-month-old infants born in 1989–1991 were recruited at well-baby clinics in Turku, Finland. All participants were of European descent. At the age of seven months, 1062 infants (56.5% of the eligible age cohort) were randomly allocated into a dietary intervention (*n* = 540) or control (*n* = 522) group (Supplemental Fig. 1S1). The cohort additionally included a pilot group (*n* = 45). The intervention group families have received dietary counseling at least twice a year (described in more detail^15^).

The first post-intervention follow-up of the participants was conducted when they were aged 26 years^16^. Of the cohort (*n* = 1116), 1072 were invited to participate, and of these, 551 provided follow-up data (51%; intervention *n* = 263 vs. control *n* = 288, Supplemental Fig. 1S1). Extensive attrition analyses have been published previously^16^. In brief, the participants and non-participants of the follow-up were similar in terms of their studied dietary components, smoking behaviour, physical activity, BMI, blood pressure, and serum lipids.

The STRIP study has been approved by the Ethics Committee of the Wellbeing Services County of Southwest Finland (Approval code: ETMK: 51/1801/2014. Approval date: 20 May 2014. Clinical Trial Registration: http://www.clinicaltrials.gov01/05/1990, Unique identifier: NCT00223600). Written informed consent was obtained from parents at study entry and from the participants at the ages of 15, 18, and 26 years. This study uses the cross-sectional post-intervention follow-up data from participants who gave faecal samples (*N* = 357; Supplemental Fig. 1S1). The final study sample consists of those included in physical activity groups, described below (*N* = 302, female 176, male 126 Table 1). Reporting followed Strengthening The Organization and Reporting of Microbiome Studies (STORMS) guideline, a reporting checklist for human microbiome research (Supplemental Table 1S2)^17^. All methods were performed in accordance with the relevant guidelines and regulations.

**Table 1:** Characteristics of the participants in the active and inactive LTPA groups.

	Active	Inactive	*P*-value
	(*N* = 219)	(*N* = 83)	
Sex^1^ [n (%)]			0.99
Female	128 (58.4)	48 (57.8)	
Male	91 (41.6)	35 (42.2)	
STRIP study group^1^[n (%)]			0.48
Control	112 (51.1%)	38 (45.8%)	
Intervention	107 (48.9%)	45 (54.2%)	
Antibiotics^1^ (*n* = 296) [n (%)]			0.19
No	192 (87.7%)	77 (92.8%)	
Yes	23 (10.5%)	4 (4.8%)	
Missing	4 (1.8%)	2 (2.4%)	
BMI^2^ (kg/m^2^)	24.1 (3.74; 14.2–43.1)	24.1 (4.65; 17.2–44.3)	0.95
Waist^2^ (cm)	79.3 (10.1; 54.1–127)	81.5 (13.8; 59.8–123)	0.19
Cardiorespiratory fitness^2^ (*n* = 104) (ml/kg/min)	40.3 (6.64; 20.9–65.4)	34.4 (5.86; 21.4–51.9)	**< 0.001**
Heavy drinking (6 or more doses) ^1^			**< 0.001**
2 times per week or more	0 (0%)	7 (8.4%)	
Once a week	8 (3.7%)	7 (8.4%)	
2–3 times per month	47 (21.5%)	15 (18.1%)	
Once a month	54 (24.7%)	12 (14.5%)	
2–6 times per year	73 (33.3%)	31 (37.3%)	
Less frequently or never	33 (15.1%)	10 (12.0%)	
Missing	4 (1.8%)	1 (1.2%)	
Smoking status ^1^ (daily smoker)			0.23
No	208 (95.0%)	75 (90.4%)	
Yes	11 (5.0%)	8 (9.6%)	
Current working status^1^			0.07
Working full-time	101 (46.1%)	27 (32.5%)	
Working, but studying as well	25 (11.4%)	9 (10.8%)	
Studying full-time	62 (28.3%)	27 (32.5%)	
Unemployed or temporarily laid off	9 (4.1%)	7 (8.4%)	
Disability support pension	0 (0%)	1 (1.2%)	
Stay-at-home mom or dad	4 (1.8%)	0 (0%)	
Other	18 (8.2%)	12 (14.5%)	
Occupational physical workload^1^			0.07
Primarily desk work	74 (33.8%)	19 (22.9%)	
Walk a fair amount at work	61 (27.9%)	20 (24.1%)	
Walk, lift things, take the stairs or go uphill a lot at work	41 (18.7%)	19 (22.9%)	
Physically strenuous job	5 (2.3%)	6 (7.2%)	
Missing	38 (17.4%)	19 (22.9%)	
Dietary factors (*n* = 280)	*N* = 203	*N* = 77	
Diet score^2^ *	19.2 (4.74; 6.00–32.0)	16.9 (5.43; 5.00–30.0)	**0.001**
Fibre-rich grain products^2^ (g/d)	75.3 (42.0; 0-218)	61.3 (40.6; 0-163)	**0.01**
Fruit and berries^3^ (g/d)	151 (181.6; 0-694)	83.6 (131.5; 0-885)	**< 0.001**
Vegetables, pulses and sprouts^2^ (g/d)	231 (134; 28.0-759)	176 (106; 0-606)	**< 0.001**
Fish^3^ (g/d)	18.8 (42.6; 0-334)	12.3 (37.4; 0-120)	0.74
Nuts and seeds^3^ (g/d)	1.00 (12.8; 0-110)	0 (0.8; 0-35.7)	**< 0.001**
Vegetable-oil based fats^2^ (g/d)	18.4 (13.4; 0-76.2)	15.6 (10.4; 0-50.2)	0.066
Low-fat unsweetened dairy^3^ (ml/d)	129 (302.7; 0-1630)	62.5 (215.4; 0-1020)	**0.011**
Red and processed meat^***3***^ (g/d)	86.6 (119.9; 0-582)	72.5 (100.4; 0-375)	0.20
Sugar sweetened beverages^3^ (ml/d)	42.5 (132.5; 0-875)	82.5 (225.0; 0-1400)	**0.05**
Desserts^3^ (g/d)	19.7 (39.3; 0-301)	21.1 (35.7; 0-296	0.55
Salty snacks^3^ (g/d)	0 (0.00; 0-43.5)	0 (0.00; 0-18.8)	0.26
Energy intake^2^ (kcal/d)	2130 (592; 841–4360)	1860 (514; 952–4340)	**< 0.001**

^1^For categorical variables the differences between the LTPA groups tested with chi-squared test.

^2^Values for normally distributed continuous variables are reported as Mean (SD; range) and the differences between the LTPA groups tested with 2 sample t-test.

^3^Values for non-normally distributed continuous variables are reported as Median (Interquartile; range) and the differences between the LTPA groups tested with Wilcoxon test.

The reported p-values are not corrected for multiple testing.

*A higher value indicative of a healthier diet.

### Physical activity

#### Physical activity assessment

LTPA was assessed by a self-administered questionnaire^18^. In the questionnaire, three multiple-choice questions were used to assess the frequency, duration, and intensity of habitual LTPA. The choices for the habitual LTPA frequency were (1) less than once per month, (2) once per month, (3) 2 to 3 times a month, (4) once a week, (5) 2 to 6 times a week, and (6) once a day. The choices for the duration were (1) 20 min, (2) 20–40 min, (3) 40–60 min, and (4) 60 min. For the intensity, the choices were (1) never sweating and becoming breathless, (2) light sweating and becoming breathless, and (3) severe sweating and being breathless. LTPA was calculated as a multiple of the resting metabolic rate (metabolic equivalent of Task [MET] h/wk) by multiplying the frequency, mean duration, and mean intensity of weekly LTPA.

The validation of the questionnaire was published previously^19^. In brief, an experimental study included 45 adults (age range 23 to 55 years, 48% females) who filled in the questionnaire, and their physical activity was measured with accelerometers and pedometers for 1 week^19^. The MET-index and its components (i.e., intensity, frequency, and duration of physical activity) correlated significantly with accelerometers (*r* = 0.26 to 0.40) and pedometers (*r* = 0.30 to 0.39)^19^. In addition, the applied questionnaire has been used in previous studies^20^ and the leisure time physical activity correlates moderately with maximum oxygen uptake (VO_2_ max) (*r* = 0.49–0.53) as well^21^.

#### Formation of physical activity groups

We used a previously described cut-off point^18^ and current PA guidelines to define the physical activity groups^22^. The guidelines recommend 2.5 h aerobic PA of moderate intensity (MET 3.0-5.9 mean ~ 4.5), and two times muscle-strengthening activity weekly, which was settled to be 0.5 h each session, with moderate intensity^22^ Based on this, the cut-off point for the active group was set as 16 MET h/wk [(4.5 MET x 2.5 h) + (4.5 MET x 1.0 h) = 15.75; ~16 MET h/wk]. The lower cut-off point of 5 MET h/wk, corresponds to ~ 1 h of moderate-intensity PA weekly^18^. Participants who had an LTPA level ≤ 5 MET h/wk, formed a group hereafter called the inactive group (*N* = 83) and participants who were estimated to meet the minimum of the current PA guidelines (16 MET h/wk) formed a group hereafter called the active group (*N* = 219). This was the final sample for statistical analyses without accounting for missing values in the covariates (see below Statistical Analyses).

### Gut microbiota

#### Faecal sample collection and storage

The gut microbiota of the STRIP participants was assessed for the first time in the 26-year follow-up study and altogether 357 26-year-old individuals from the original cohort provided a faecal sample successfully (described in more detail^23,24^. The participants collected the faecal samples at home and mailed them to the study centre. The participants were guided to collect a small amount of faecal material, about 500 mg, in an OMNIgene^®^ GUT (DNA Genotek, Ottawa, ON, Canada) collection tube. They were then guided to homogenise the sample with 30 s of vigorous shaking and to mark the tube with the date and time of sampling. The participants were instructed to mail the samples to the laboratory as soon as possible. The collection tubes include a stabilizing solution that guarantees DNA integrity in typical ambient temperature fluctuations and stability at room temperature for 60 days. Information on antibiotics and probiotics use, gastroenteritis, and faecal composition was collected.

#### DNA sequencing and bioinformatics

Three samples were excluded due to low sample quality, and ten samples had unsuccessful sequencing, as described previously^23^. Sample processing and DNA extraction were performed as previously reported^23^. Briefly, the samples were homogenised with light mixing, and bacterial DNA was extracted using the GXT Stool Extraction Kit VER 2.0 (Hain Lifescience GmbH, Nehren, Germany) from 200 to 250 µL of the sample solution according to manufacturer instructions with the exception that cell lysis was enhanced by an additional bead-beating with MOBIO PowerLyzer 24 Bench Top Bead-Based Homogenizer (MO BIO laboratories, Inc, Carlsbad, CA, USA)^23^. The Qubit dsDNA HS Assay kit and Qubit 2.0 fluorometer (Thermo Fisher Scientific, Waltham, MA USA) were used for the measurement of the DNA concentrations. The DNA was kept at -75 °C. Microbial profiles were analysed with 16 S rRNA gene sequencing. The variable region V4 was amplified with custom-designed dual-indexed primers and sequenced using an Illumina MiSeq system as described previously^23^. The DNA extraction and sequencing were performed at the University of Turku. Every sequencing batch additionally included a positive, in-house generated plasmid-mix control and a negative water control. The samples were sequenced in five batches, and the inactive and active individuals were distributed evenly between batches (χ^2^-test *p* = 0.31).

The Illumina BaseSpace platform was used to demultiplex the sequencing data and clip the sequence adapters, primers, and barcodes. The DADA2 pipeline implemented in the *dada2* R package was used to convert the raw sequencing data into amplicon sequence variants (ASV)^25^. The demultiplexed fastq files were filtered and trimmed, each sample was dereplicated. Function *dada* was applied using the default parameters and forward and reverse reads were merged. The function *isBimeraDenovo* was used to filter out chimeric sequences. Taxonomy was assigned with function *assignTaxonomy*. The Ribosomal Database Project database (RefSeq- RDP16S v2 May2018) was used to supplement the NCBI RefSeq 16 S rRNA database for the taxonomic classification. The pre-processing resulted total of 6591 distinct ASVs from 291 bacterial genera and 20 bacterial phyla. The total read counts from the 16 S rRNA gene sequencing within the study population were min: 11.8 k, max: 839 k, median: 160 k.

### Diet score

Information on diet was assessed with a food diary before the follow-up visit on four consecutive days, including 1–2 days on weekend^16^. Portion sizes were estimated using household measures or a food picture booklet, with details such as brand and preparation method noted. Dietary technicians reviewed and completed the diary during study visits. Data from the food diary were entered into Micro-Nutrica^®^ software, capable of analysing over 4000 foods and dishes, to calculate food and nutrient intake, incorporating 66 nutrient values^23^.

A diet score reflecting an overall healthy diet was calculated based on the food diary data^15^. The diet score is adapted from Nettleton et al.^26^ and improved for better suitability for the Finnish population^15^. Other similar diet scores have been previously used to assess dietary patterns^27^ and higher scores have been associated with health outcomes, such as lower fasting glucose and insulin levels^26^, lower coronary heart disease, and stroke risk^28^. In our previous study, the STRIP intervention group had a higher diet score value^15^.

Higher consumption of fibre-rich grain products, fruits and berries, vegetables, fish, nuts and seeds, and low-fat, unsweetened dairy and lower consumption of red and processed meat, sugar-sweetened beverages, salty snacks, and desserts loaded to a higher dietary index score (Supplemental Table 2S3). Food intake within each food group was calculated in grams/energy intake and quantified into quartiles based on participants’ intake. For each study participant, the points were given by quartiles. For favourable foods, ascending values by quartiles (0 to 3, with the highest consumption meaning 3 points) were assigned, whereas for unfavourable foods, descending values by quartiles were given (3 to 0, the lowest consumption meaning 3 points). The sum of these values resulted in an overall diet score (range 0–33), with higher scores indicating a healthier diet based on guidelines^15,29,30^ (Supplemental Table 2S3).

### Other characteristics

At the follow-up study visit, height, weight, waist circumference, blood pressure, and cardiorespiratory fitness were measured as described previously^16,31^. A self-administered questionnaire was used to assess smoking status, frequency of consuming 6 or more portions of alcohol at a time, probiotics, antibiotics use (yes/no) during the prior 6 months as well as current health status. Study subjects also reported their occupation (1 = Working full-time; 2 = Working, but studying as well; 3 = Studying full-time; 4 = Unemployed or temporarily laid off; 5 = Disability support pension; 6 = Stay-at-home mom or dad; 7 = Other) and the occupational physical workload (1 = My work is primarily desk work and I don’t walk very much at work (e.g. office work in front of a desk); 2 = I walk a fair amount at work, but I don’t have to lift or carry heavy burdens (e.g. shop assistant’s work, light industrial work, mobile office work); 3 = In my work I have to walk, lift things, take the stairs or go uphill a lot (e.g. carpenter’s work, machine workshop, and other heavier industrial work; 4 = I have a physically strenuous job, where I have to lift or carry heavy objects, dig, shovel or pummel, etc. (e.g. forestry, heavy agricultural work, heavy construction, and industrial work)).

### Statistical analyses

All statistical analyses were performed with R (v. 4.2.1, R Foundation for Statistical Computing, Vienna, Austria; https://www.R-project.org/), with Bioconductor packages, *mia*^32^ and *vegan*^33^. The normality of the distribution of continuous variables were inspected with histograms. Differences in background characteristics between groups were tested with 2 sample t-test for normally distributed continuous variables, the Wilcoxon test for non-normally distributed continuous variables, and a chi-squared test for categorical variables. The alpha and beta diversity analyses were performed on all non-rarefied data, including rare taxa. Alpha diversity was assessed on ASV-level using the Shannon index and observed richness with the *mia* package. Group differences were tested with the Wilcoxon rank sum test. The normality of the distribution of the Shannon index and observed richness were inspected with histograms. We assessed the sensitivity of alpha diversity to sequencing depth by including sequencing depth as a covariate in a linear model analysing the association of alpha diversity with LTPA groups.

The suitability of the groups for beta diversity analysis was tested with beta dispersion using Bray-Curtis dissimilarity and PERMDISP2 from the *vegan* package. Beta dispersion represents the homogeneity of the variances of average distances to group centroid. Beta diversity was analysed in ASV-level with Permutational Multivariate Analysis of Variance (PERMANOVA) using the *adonis2* function in *vegan* and Bray-Curtis dissimilarity with 999 permutations. Principal Coordinates Analysis (PCoA) plot for visualisation was generated using *vegan.* Function *adonis2* was used to check the largest absolute coefficients in genus level driving the difference in beta diversity.

For differential abundance (DA) analysis, *DESeq2* was used at the genus level. To limit multiple comparisons, taxa with prevalence over 10% were included in the differential abundance analysis. Filtering rare taxa may reduce lab-to-lab variability between samples and dimensionality without information loss or impact on scientific conclusions^34^. P-values were adjusted for multiple comparisons with the Benjamini-Hochberg method in DA analysis and we consider findings with adjusted *p* < 0.05 (referred to as adj.p) as statistically significant. If significant findings with either alpha diversity, beta diversity, or DA analysis were found, those analyses would be repeated with the diet score included in the models with the following formula:

alpha diversity/beta diversity/genera abundance ~ LTPA group + diet score.

Antibiotic use may associate with gut microbiota composition. To address for this potential confounder, we conducted sensitivity analysis by excluding those treated with antibiotics or missing antibiotic use data and repeated all the analyses (overall *N* = 269, with diet score *N* = 250). We don’t think that antibiotics used are a common cause for LTPA and gut microbiota, and hence we did not include binary antibiotic use as a covariate. The R scripts are available in the Supplementary Methods.

## Results

Of the participants, 83 (27.5%) belonged to the inactive LTPA group (≤ 5 MET h/wk), and 219 (72.5%) were in the active group. Anthropometric, lifestyle, and dietary characteristics in the groups at age 26 years are shown in Table 1. Participants in the inactive group had poorer cardiorespiratory fitness (VO_2_ max), lower energy intake, and a less healthy diet, but there were no differences in antibiotic use, smoking, current working status, occupational physical workload, or sex distribution between the groups. In addition, the groups had a difference in the frequency of heavy alcohol intake (Table 1).

### Associations between physical activity and gut microbiota

#### Alpha diversity

**Fig. 1:**
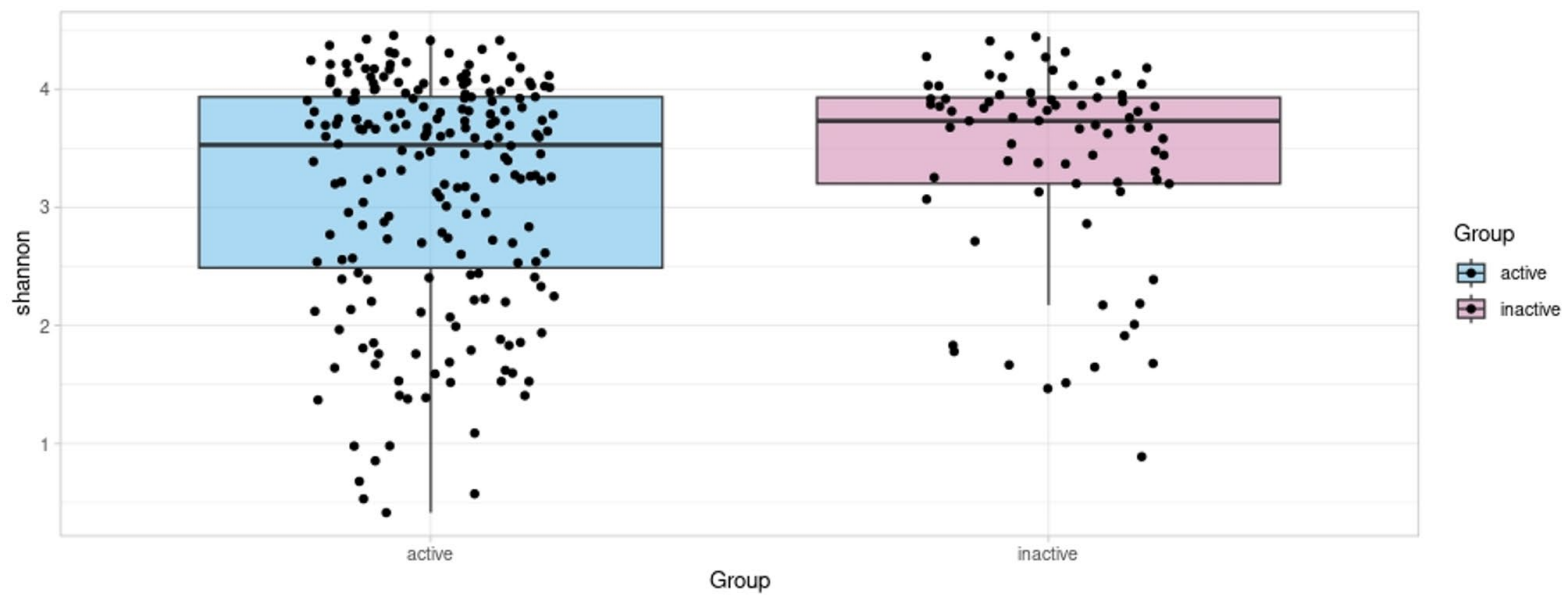
Shannon diversity in the groups (blue = active, pink = inactive) based on Shannon index (active: mean = 3.17; inactive, mean = 3.37).

We found no significant differences in alpha diversity assessed as Shannon index and observed richness between the active and inactive groups (Wilcoxon *p* = 0.08 and *p* = 0.12, respectively; Fig. 1). We studied whether the sequencing depth affects the association between alpha diversity and LTPA groups. There was no difference in alpha diversity between LTPA groups after sequencing depth was included (data not shown).

### Beta diversity

**Fig. 2:**
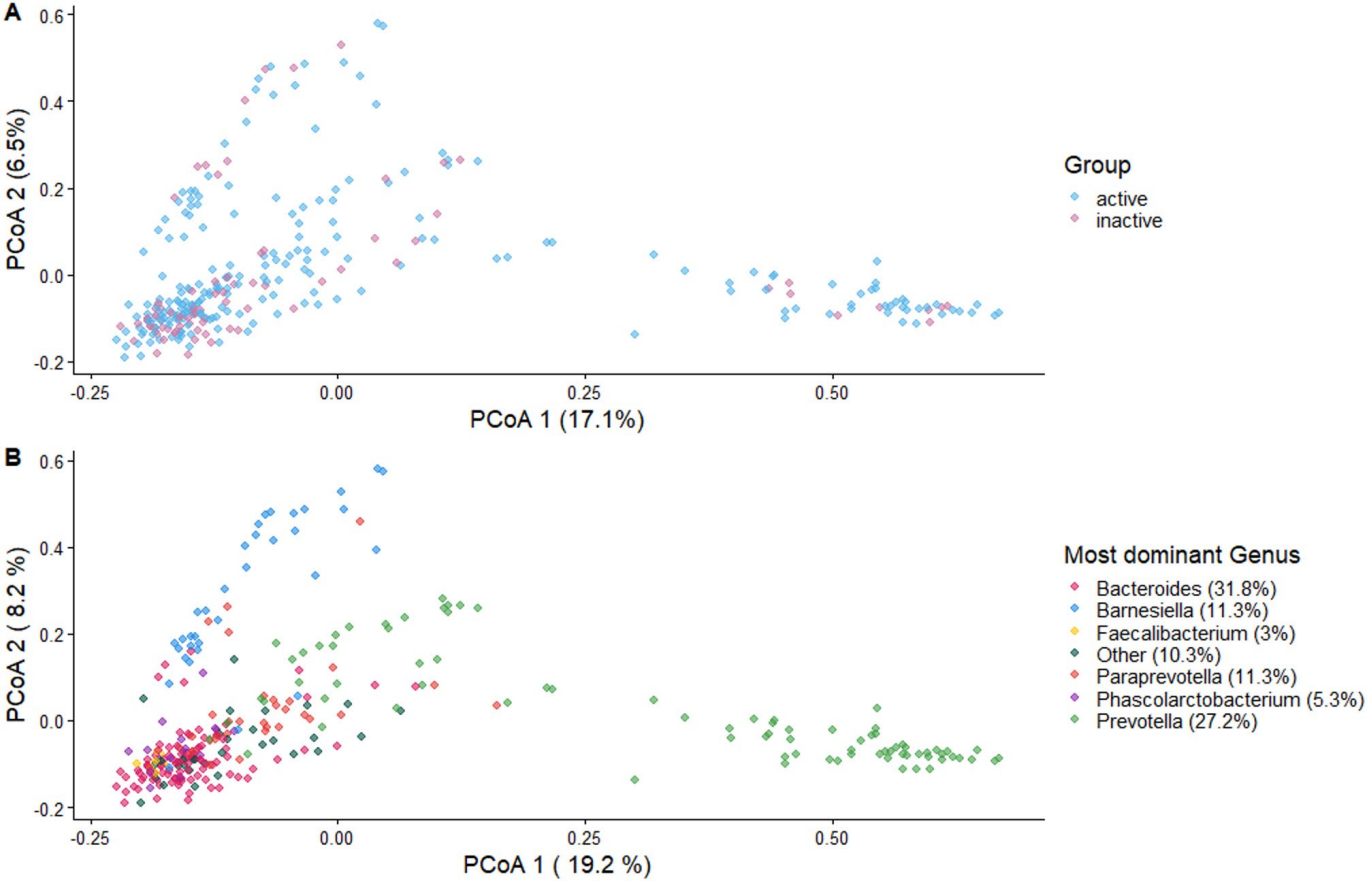
(**A**) Beta diversity at the ASV-level amongst the physical activity groups based on Bray-Curtis dissimilarities (blue dots: active group, pink dots: inactive group). (**B**) The most dominant genus for each participant. The colours indicate the group each individual belongs to as detailed in the legend.

**Fig. 3:**
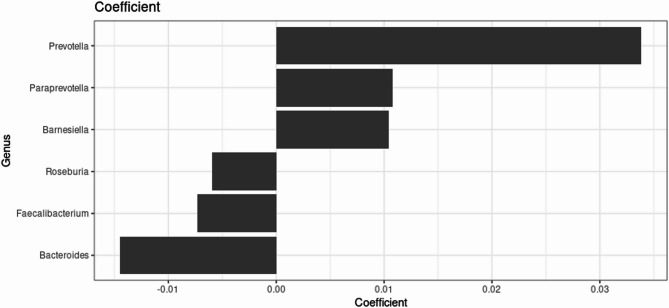
Highest absolute coefficients of the linear model in Permanova. This describes the top genera driving the difference in Bray-Curtis dissimilarity between the physical activity groups (< 0 = inactive, > 0 = active group).

Beta dispersion was similar between the LTPA groups (*p* = 0.06), which indicates that LTPA groups were equally homogeneous in their distances to the group centroid and suitable for beta diversity analysis. The PERMANOVA analysis suggested that the observed community compositions differ between the groups regarding Bray-Curtis dissimilarity (R^2^ = 0.005, *p* = 0.044) on ASV-level. This indicates that the groups on average differ by their microbial community composition when accounting for taxa abundances. The Principal Coordinates Analyses the PCoA plot (Fig. 2) based on the Bray-Curtis dissimilarity measure visualises the community composition. The strongest associations were found for the genera *Prevotella*,* Paraprevotella*,* Barnesiella*,* Bacteroide*s, *Roseburia*, and *Faecalibacterium* (Fig. 3). Accordingly, an elevated abundance of genus *Bacteroides* in the inactive group and certain *Prevotella* and *Paraprevotella* species in the active group drive the difference in beta diversity.

### Differential abundance analyses

**Fig. 4:**
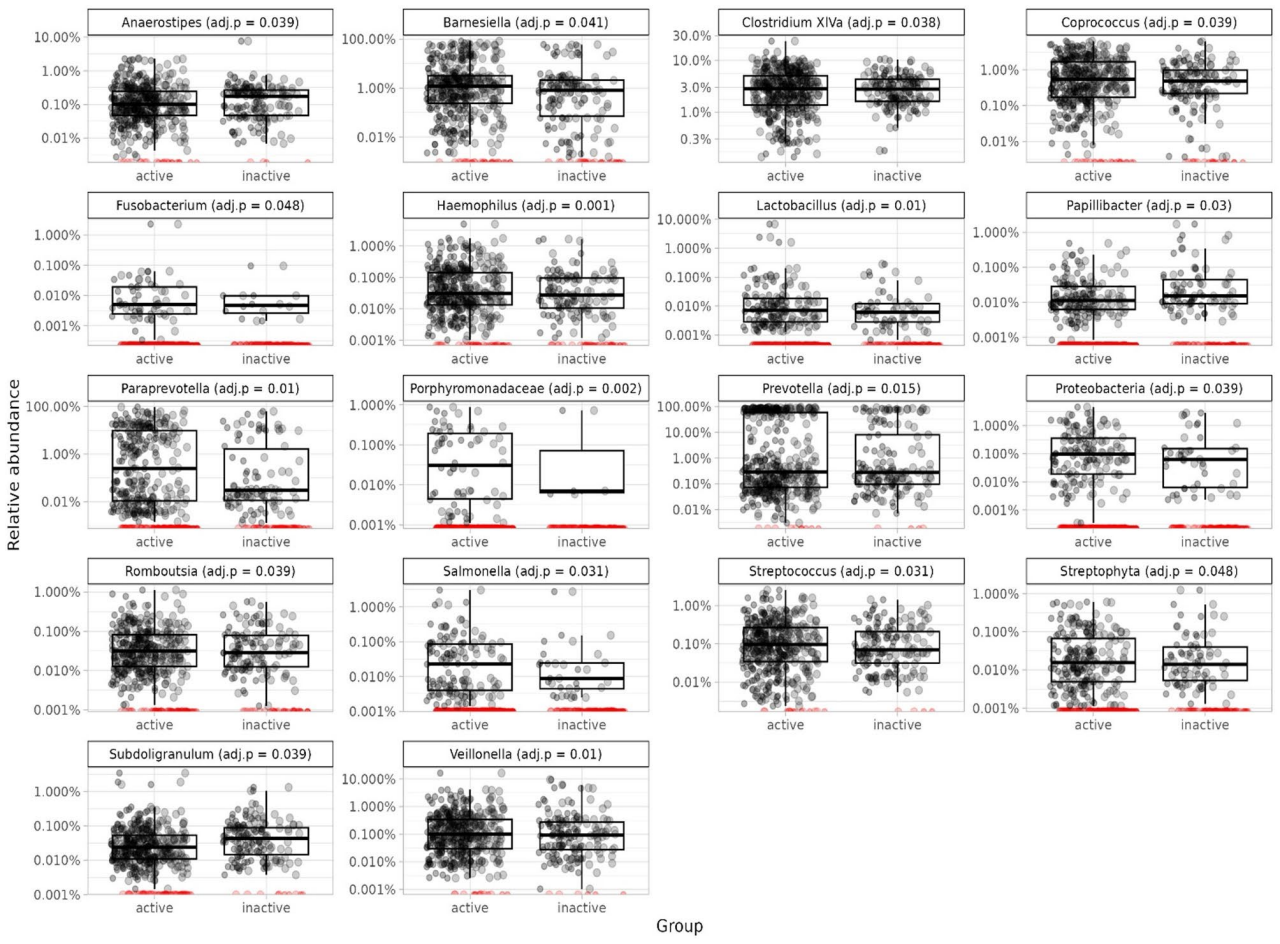
Relative abundance of those genera that were identified as differentially abundant in *DESeq2* analysis between active and inactive groups before adding diet to the model (black dots: abundance > 0, red dots: abundance = 0). The adjusted p values (adj.p) are obtained from the *DESeq2* analysis.

The *DESeq2* analysis identified 18 genera that differed between the active and inactive LTPA groups (Fig. 4, Supplemental Table 3S4). The inactive group had a higher abundance of *Papillibacter* (adj.*p* = 0.030) and *Subdoligranulum* (adj.*p* = 0.039). The active group had a higher abundance of *Haemophilus* (adj.*p* < 0.001), *Porphyromonadaceae* (adj.*p* = 0.002*)*,* Lactobacillus* (adj.*p* = 0.010), *Paraprevotella* (adj.*p* = 0.010), *Veillonella* (adj.*p* = 0.010), *Prevotella* (adj.*p* = 0.015), *Salmonella* (adj.*p* = 0.031), *Streptococcus* (adj.*p* = 0.031), *Clostridium XlVa* (adj.*p* = 0.038), unknown *Proteobacteria* (adj.*p* = 0.039), *Romboutsia* (adj.*p* = 0.039), *Coprococcus* (adj.*p* = 0.039), *Anaerostipes* (adj.*p* = 0.039), *Barnesiella* (adj.*p* = 0.041), *Fusobacterium* (adj.*p* = 0.048), and *Streptophyta* (adj.*p* = 0.048).

### Adjustment for diet

**Fig. 5:**
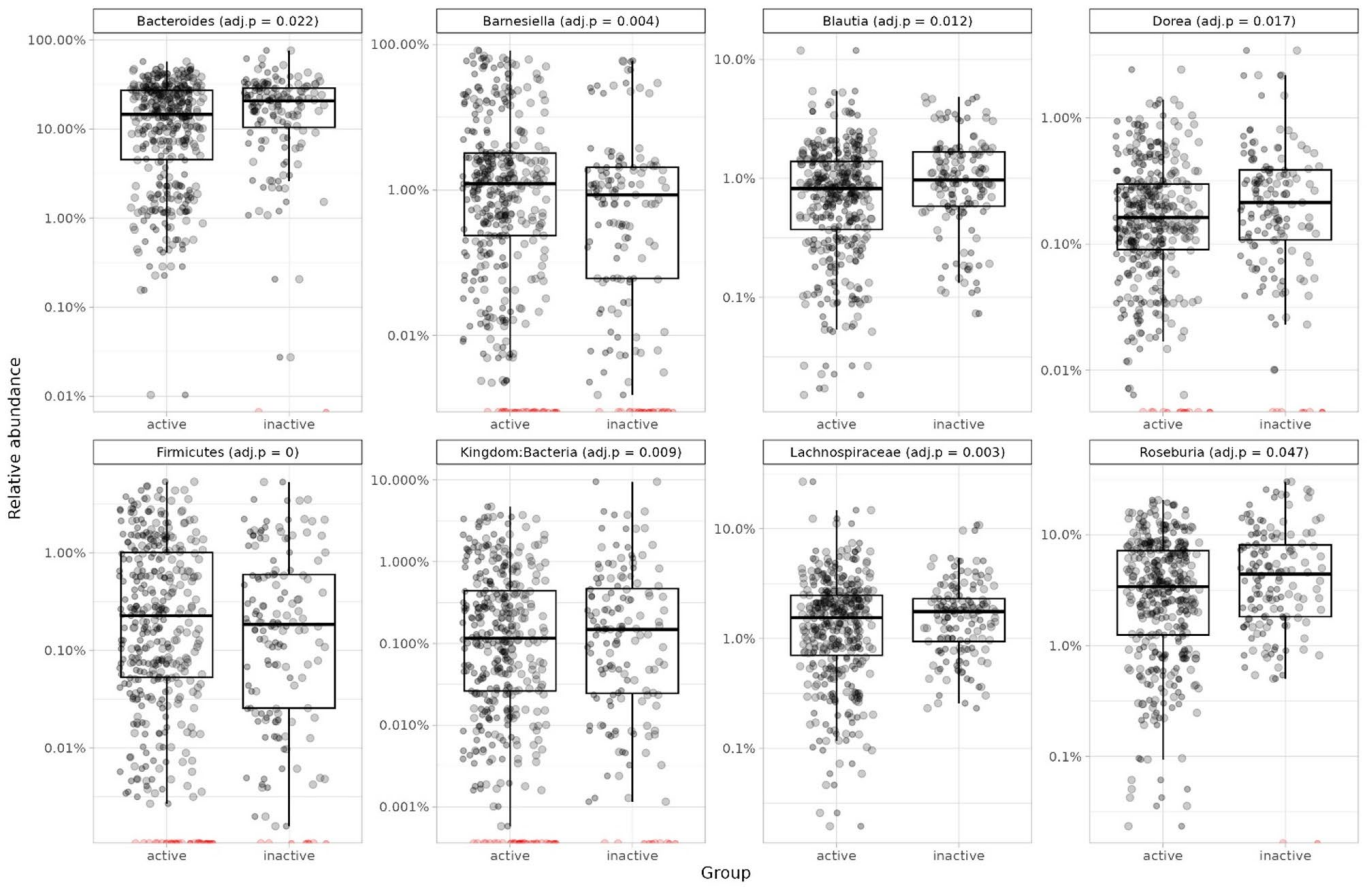
Relative abundance of those genera that were identified as differentially abundant in active and inactive groups after diet was added to the model (black dots: abundance > 0, red dots: abundance = 0). ). The adjusted p values (adj.p) are obtained from the *DESeq2* analysis.

The PERMANOVA and DA analyses were repeated with the diet score included as a covariate (data available for analyses: *N* = 280, active = 203, inactive = 77). After the adjustment for the diet score, the association of the LTPA group with beta diversity was diluted (R^2^ = 0.005, *p* = 0.097). Of note, the association of diet score with beta diversity was significant, while still including the LTPA group in the model (R^2^ = 0.009, *p* = 0.003). The diet score adjusted DA analysis with *DESeq2* identified 8 genera that differed between the groups, all but *Barnesiella* (adj.*p* = 0.004), distinct from those previously identified without diet score. The active group had a higher abundance of unknown *Firmicutes* (adj.*p* = 0.0002), unknown *Lachnospiraceae* (adj.*p* = 0.003), and unknown *Bacteria* (adj.*p* = 0.009). The inactive group had a higher abundance of *Blautia* (adj.*p* = 0.012), *Bacteroides* (adj.*p* = 0.022), *Roseburia* (adj.*p* = 0.046), and *Dorea* (adj.*p* = 0.017) (Fig. 5).

### Sensitivity analyses

We conducted a sensitivity analysis by removing those individuals who had used antibiotics or had missing antibiotic information from the models (data available *N* = 269, active = 192, inactive = 77). The results remained essentially the same, but the significant differences between LTPA groups in beta diversity were preserved after adjustment with diet.

We found no significant differences in alpha diversity assessed as Shannon index and observed richness between the active and inactive groups (Wilcoxon *p* = 0.11 and *p* = 0.14, respectively). In the PERMANOVA analysis, the observed community composition differed between the LTPA groups regarding Bray-Curtis dissimilarity (R^2^ = 0.008, *p* = 0.012) on ASV-level. Lastly, we repeated the PERMANOVA and DA analyses with diet score added to models without those treated with antibiotics (*N* = 250, active = 178, inactive = 72). The PERMANOVA analysis remained significant between the LTPA groups and community composition regarding Bray-Curtis dissimilarity (R^2^ = 0.007, *p* = 0.023), even with diet included in the model. The results of *DESeq2 *analyses remained mostly similar compared to the previous analyses as described in detail in Supplementary Information (Supplemental Fig. 2S5 without adjustment for the diet and Supplemental Fig. 3S6 with adjustment for the diet).

## Discussion

Previous studies have associated vigorous PA, e.g., exercise interventions instructing running or cycling 30–60 min 3–6 times per week, sprint interval training, or athletic background, with altered GM composition^5,35,36^. The present study used a relatively large population-based cohort of young adults, to explore associations of LTPA with differences in GM composition. We compared the GM of individuals with at least a moderate level of LTPA, meeting or exceeding the minimum recommended physical activity, to those with a low LTPA level. We show that LTPA is associated with GM composition, but not alpha diversity when comparing inactive individuals with those meeting the estimated recommended amount of PA. In DA analyses, we found differences in the genera abundances, such as higher *Lactobacillus*,* Veillonella*,* Prevotella*, and *Barnesiella* abundances in the active group compared with those in the inactive group. The studied groups differed not only in their physical activity but also in their dietary choices, with the more active group displaying a healthier diet. When further adjusting for diet score, the association of PA and beta diversity diluted but was redeemed after sensitivity analysis excluding those treated with antibiotics. It suggests that associations of PA with GM are likely, at least partly, confounded by diet and antibiotic use. Since not all previous studies investigating the association of PA and GM have taken into account diet, we discuss our findings in light of the previous literature with and without adjusting for diet.

Recent reviews investigating PA and GM^1,10^ have shown an increased abundance among several taxa, including *Bifidobacterium*,* Faecalibacterium prausnitzii*,* Prevotella*,* Lactobacillus acidophilus*,* Haemophilia*,* Proteobacteria*,* Verrucomibrobia* and *Veillonella*, with higher PA. In line, we observed a positive association of PA with a higher abundance of *Prevotella*,* Haemophilus*,* Lactobacillus*, and *Veillonella*, although these findings were driven by differences in diet. Furthermore, the largest differences in community composition between inactive and active were driven by the genera *Prevotella*,* Paraprevotella*,* Bacteroides*, and *Faecalibacterium*, among which the *Bacteroides* and *Faecalibacterium* were more abundant in the inactive group. These associations were diluted after adjustment for diet. However, when we excluded those treated with antibiotics, the LTPA groups had difference in the community composition even after adjustment for diet. This indicates that in those subjects with no recent history of antibiotic use, LTPA is related to community composition even after accounting for diet.

In contrast to previous findings^37^, in our study, *Faecalibacterium –* often considered beneficial for health^38^ - was less abundant in the active group, even though they consumed more fruits and vegetables. This association was found before adjustment for diet and after adjustment in those not treated with antibiotics. Plant-based diet and exercise have been previously associated with increased *Faecalibacterium*^38^. One potential explanation for decreased *Faecalibacterium* in our study may be meat consumption: the active group consumed more meat, which has been associated with a decrease in *Faecalibacterium* abundance^8^. The second potential explanation is that vigorous PA induced gastrointestinal permeability, inflammation, and oxidative stress what could lead to a decrease in *Faecalibacterium prausnitzii*, which is sensitive to oxidative conditions^39,40^.

Similarly to our study, a decrease in the genus *Bacteroides* and an increase in the abundance of *Prevotella* has been associated with higher PA, in a study where diet had been controlled and participants with antibiotic use excluded^41^. Šoltys et al.^41^ found that among elderly men the *Bacteroides* to *Prevotella* ratio was one of the strongest indicators for separating physically active individuals from lower activity level controls, along with cardiorespiratory fitness and BMI. In cyclists, a higher abundance of *Prevotella* correlated with higher reported exercising time^42^. Furthermore, athletes who consume more carbohydrates and fibre seem to have higher levels of *Prevotella*, which is favorably connected with amino acid and carbohydrate metabolic pathways, including increased branched-chain amino acid metabolism that may reduce PA induced fatigue^1^. The *Prevotella*-dominated microbiota can utilise more fibre from diet and produce 2–3 times more propionate than the *Bacteroides-*dominated microbiota^43^. In our study, we found similar difference in community composition, where a decrease in *Bacteroides* and an increase in *Prevotella* abundance were associated with PA, even when accounting for diet and antibiotic use.

Our study corroborates that higher PA level is associated with an increase in the abundance of *Veillonella* and *Lactobacilli* genera^1,10^. However, in our study, the associations between PA groups with *Veillonella* and *Lactobacillu*s were confounded by diet. Interestingly, in previous studies these genera have been linked to properties that can improve athletic abilities. In Scheiman et al.^44^ study where the diet was controlled, increased abundance of the genus *Veillonella* was found post-marathon, and the species *Veillonella atypica* was linked to enhanced physical performance by converting the exercise-induced lactate into propionate. In mice, it has been linked to improved treadmill performance^44^. Additionally, *Veillonella atypica* may improve muscular strength and mitochondrial efficiency by metabolising inorganic nitrate to nitric oxide and related nitrogen oxides^45,46^. In their study, O’Donovan et al.^47^ associated an increase in *Lactobacilli* with moderate to high dynamic sports that require higher cardiac output, with information on diet. Widely studied *Lactobacilli* are considered beneficial for health and are commonly used as probiotics. *Lactobacillus* may reduce sensitivity to inflammation and help maintain redox balance in individuals while engaging in PA^48^. Given the cross-sectional design of our study, we cannot determine the causality of the associations between PA and these genera. However, prior research suggests that the relationship is likely bidirectional^4^.

In our study, the genus *Barnesiella* was the only genus with a higher abundance in the active group both before adjustment to the diet, after adjustment, as well as after sensitivity analysis for antibiotics. The previous research regarding the genus *Barnesiella* in the context of PA or fitness is scarce and inconsistent. *Barnesiella* has been associated with less physical activity in older adults with insomnia in a study that controlled for diet^49^. However, another study found a higher *Barnesiella* abundance in high-functioning older adults^50^. In a rodent study, a decrease in *Barnesiella* was connected to the age-related reduction in body lean mass^51^. *Barnesiella* has also been associated with the production of acetate, a SCFA produced by gut microbes, which might decrease visceral fat^52^. Overall, it seems that our results are partly differing from the observations made in older adults and the elderly. Further research is needed to explore whether the association between *Barnesiella* and PA is age-dependent.

Our results additionally show that associations between LTPA and GM remain relatively similar even after exclusion of participants with prior antibiotic use. Interestingly, the community composition was different between active and inactive even when accounting for diet in this subsample. We believe that this indicates that antibiotic use is not a major confounder in the context of LTPA and microbiota in young adults drawn from the general population. It has been shown in a population-based cohort that cumulative, long-term use of antibiotics leads to more pronounced differences in GM composition^53^. Additionally, narrow-spectrum antibiotics are associated with more limited change in GM, especially when the follow-up is longer^53-55^. We had self-reported data on any antibiotic use. Further studies with detailed information on frequency, duration and antibiotic groups used could help to show whether long-term, frequent or cumulative use of antibiotics could be a mediator or moderator between PA and GM.

The key strength of our study was a relatively large population-based cohort of young adults, with detailed information on diet. That said, this study has some potential limitations. Firstly, data on PA were collected with a self-administered questionnaire, which may induce reporting bias and does not reflect the type of PA. The assessed PA data were limited to leisure time and did not include e.g., active commuting or occupational PA, which could have affected the PA volume in some participants. However, the current working status and the occupational physical workload did not significantly differ between the LTPA groups. Furthermore, those in the active group had better cardiorespiratory fitness, suggesting that the reported leisure time PA reflects the participants’ PA levels. Secondly, there was a variety of PA levels in the active group, consisting of moderate and highly active individuals. It is therefore possible that the small proportion of very active individuals drives the differences in GM composition. Thirdly, GM composition was determined using faecal samples, which may not give the full understanding of the GM composition in the intestine. Fourth, using 16 S ribosomal RNA gene amplicon sequencing is a reliable and affordable method to determine the overall composition of the microbiome, but the resolution does not reach species or strain levels, and it may underestimate GM diversity. Fifth, the study interpretations are limited by focusing on young, adults of European descent.

This study explored whether GM composition differs between individuals meeting the minimum amount of recommended PA and inactive individuals. Our study suggests that PA may be associated with overall differences in GM composition and increased abundance of specific genera considered health-beneficial, such as the *Lactobacillus.* Collectively, physical activity may be linked with potentially health-promoting properties of gut microbiota, although diet may partially drive the associations. Our study highlights that it is important to account for the differences in diet when studying PA and GM. Future studies applying more in-depth characterised GM data may reveal specific species linked with PA and shed light on the underlying mechanisms interconnecting PA and GM.

## Electronic supplementary material

Below is the link to the electronic supplementary material.

Supplementary Material 1

## Data Availability

The dataset supporting the conclusions of this article were obtained from the STRIP study. The STRIP dataset comprises health-related participant data, and its use is therefore restricted under the regulations on professional secrecy (Act on the Openness of Government Activities, 612/1999) and on sensitive personal data (Personal Data Act, 523/1999, implementing the EU data protection directive 95/46/EC). Due to these legal restrictions, the data from this study cannot be stored in public repositories or otherwise made publicly available. Data sharing outside the STRIP research group requires a data-sharing agreement as part of research collaboration. Investigators interested in research collaboration may contact the chairman of the STRIP steering group (Prof. Olli Raitakari, olli.raitakari@utu.fi, University of Turku, Turku, Finland).
