# Supplementary material for: Association of leisure time physical activity with gut microbiota composition in early adulthood
Source: Sci Rep. 2025 Jun 4;15:19697. doi: 10.1038/s41598-025-02287-2 (PMC12137592; doi:10.1038/s41598-025-02287-2)
Supplement: Supplementary file 1 — Supplementary Material 1 [file 41598_2025_2287_MOESM1_ESM.docx]

**Association of Leisure Time Physical Activity with Gut Microbiota Composition** **in Early Adulthood**

**Hanna-Mari Boelius ^1;2*^; Anna-Katariina Aatsinki^1,3*^; Marja A. Heiskanen^1,2*^; Eero A. Haapala^4,5^; Eveliina Munukka^6,7^; Juha Mykkänen^1,^­^2^; Noora Kartiosuo^1,2,8^; Leo Lahti^9^; Anniina Keskitalo^7^; Pentti Huovinen^5,7^; Harri Niinikoski^1,10^; Jorma Viikari^11,12^; Tapani Rönnemaa^11,12^; Hanna Lagström^1,13,14^; Antti Jula^15^; Suvi P. Rovio^1,2,13^; Olli T. Raitakari^1,2,16^; Katja Pahkala^1,2,17^**

**^*^corresponding author**

Supplementary Information

*Supplemental figure 1 S1: Description of the STRIP study design.*

*Supplemental table 1 S2: STORMS checklist*

*Supplemental table 2 S3: Diet score food groups and description*

*Supplemental table 3 S4: Differential abundance analysis results.*

*Supplemental Information 1: Results after exclusion of those treated with antibiotics or missing antibiotic information.*

Supplemental figure 2 S5: Relative abundance of those genera that were identified as differentially abundant in active and inactive groups before adding diet to model (black dots: abundance > 0, red dots: abundance = 0). The adjusted p values (adj.p) are obtained from the DESeq2 analysis. The antibiotic treated excluded from the analysis (N=269).

Supplemental figure 3 S6: Relative abundance of those genera that were identified as differentially abundant in active and inactive groups after adding diet to model (black dots: abundance > 0, red dots: abundance = 0). The adjusted p values (adj.p) are obtained from the DESeq2 analysis. The antibiotic treated excluded from the analysis (N=250).

*Supplementary methods: R script*

Supplemental figure 1 S1: Description of the STRIP study design.


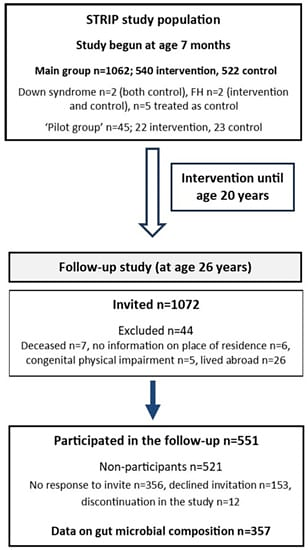


Supplemental table 1 S2: STORMS checklist

| Number | Item | Recommendation | Item  Source | Additional Guidance | Yes/ No/NA | Comments or location in manuscript |
| --- | --- | --- | --- | --- | --- | --- |
| **Abstract** | | | | | | |
| 1.0 | Structured or  Unstructured  Abstract | Abstract should include information on background,  methods, results, and conclusions in structured or unstructured format. | STORMS |  | Yes |  |
| 1.1 | Study Design | State study design in abstract. | STORMS | See 3.0 for additional information on study design. | Yes |  |
| 1.2 | Sequencing methods | State the strategy used for metagenomic classification. | STORMS | For example, targeted 16S by qPCR or sequencing, shotgun metagenomics, metatranscriptomics, etc. | Yes | Abstract, Gut microbiota , DNA sequencing and bioinformatics |
| 1.3 | Specimens | Describe body site(s) studied. | STORMS |  | Yes | Abstract, Physical activity, Formation to physical activity groups, table 1 |
| **Introduction** | | | | | | |
| 2.0 | Background  and Rationale | Summarize the underlying background, scientific  evidence, or theory driving the current hypothesis as well as the study objectives. | STORMS |  | Yes | Introduction |
| 2.1 | Hypotheses | State the pre-specified hypothesis. If the study  is exploratory, state any pre-specified study objectives. | STORMS |  | YES | Introduction |
| **Methods** | | | | | | |
| 3.0 | Study Design | Describe the study design. | STORMS | Observational (Case-Control, Cohort,  Cross-sectional survey, etc.) or Experimental (Randomized controlled trial, Non-randomized controlled trial, etc.). For a brief description of common study designs see: DOI: 10.11613/BM.2014.022  If applicable, describe any blinding (e.g.  single or double-blinding) used in the course of the study. |  | Material and Methods,  Study sample |
| 3.1 | Participants | State what the population of interest is,  and the method by which participants are sampled from that population. Include relevant information on physiological state of the subjects or stage in the life history of disease under study when participants were sampled. | STORMS | Examples of the population of interest could be:  adults with no chronic health conditions, adults with type II diabetes, newborns, etc. This is the total population to whom the study is hoped to be generalizable to. The sampling method describes how potential participants were selected from that population.  If the participants are from a substudy of  a larger study, provide a brief description of that study and cite that study.  Clearly state how cases and controls are defined.  An example of relevant physiological state might be pre/ post menopausal for a vaginal microbiome study;  examples of stage in the life history of disease could be whether specimens were collected during active or dormant disease, or before or after treatment. | Yes | Study sample |
| 3.2 | Geographic location | State the geographic region(s) where participants  were sampled from. | MIxS:  geographic location (country and/or sea,region) | Geographic  coordinates can be reported to prevent potential ambiguities if necessary. | NA | Study sample |
| 3.3 | Relevant Dates | State the start and end dates for recruitment,  follow-up, and data collection. | STORMS | Recruitment is the period in which participants are  recruited for the study. In longitudinal studies, follow-up is the date range in which participants are asked to complete a specific assessment. Finally, data collection is the total period in which data is being collected from participants including during initial recruitment through all follow-ups. | Yes | Study population |
| 3.4 | Eligibility criteria | List any criteria for inclusion and exclusion of recruited participants. | Modified  STROBE | Among potential recruited participants, how were some  chosen and others not? This could include criteria such as sex, diet, age, health status, or BMI.  If there is a primary and validation sample, describe inclusion /exclusion criteria for each. | Yes | Study population ,  MET/h/week |
| 3.5 | Antibiotics Usage | List what is known about antibiotics usage before  or during sample collection. | STORMS | If participants were excluded due to current or recent  antibiotics usage, state this here.Other factors (e.g. proton pump inhibitors, probiotics, etc.) that may influence the microbiome should also be described as well. | Yes | Other characteristics,  Table 1 |
| 3.6 | Analytic sample size | Explain how the final analytic  sample size was calculated, including the number of cases and controls if relevant, and reasons for dropout at each stage of the study. This should include the number of individuals in whom microbiome sequencing was attempted and the number in whom microbiome sequencing was successful. | STORMS | Consider use of a flow diagram (see template at <https://stormsmicrobiome>.org/figures). Also state sample size in abstract.  If power analysis was used to calculate sample size,  describe those calculations. | Yes | Study population,  Formation of physical activity groups, Supplement 1 (STRIP FLOW) |
| 3.7 | Longitudinal Studies | For longitudinal studies, state how many  follow-ups were conducted, describe sample size at follow-up by group or condition, and discuss any loss to follow-up. | STORMS | If there is loss to follow-up, discuss the likelihood  that drop-out is associated with exposures, treatments, or outcomes of interest. | NA |  |
| 3.8 | Matching | For matched studies, give matching criteria. | Modified  STROBE | "Matched" refers to matching between comparable study  participants as cases and controls or exposed / unexposed.  Indicate whether participants were individual or frequency  matched and in what ratio were they matched (e.g. 1 case to 1 control). | NA |  |
| 3.9 | Ethics | State the name of the institutional review board that  approved the study and protocols, protocol  number and date of approval, and procedures for obtaining informed consent from participants. | STORMS |  | YES | Study population,  Ethics approval |
| 4.0 | Laboratory methods | State the laboratory/center where laboratory work  was done. | STORMS | Provide a reference to complete lab protocols if previously published elsewhere such as on protocols.io. Note any  modifications of lab protocols and the reason for protocol modifications. |  | DNA sequencing |
| 4.1 | Specimen collection | State the body site(s) sampled from and how specimens were collected. | MIxS:  sample collection device or method; host body site | Use terms from the Uber-anatomy Ontology (https://www.ebi.ac.uk/ols/ontologies/uberon) to describe body sites in a standardized format. | YES | Faecal sample  collection and storage |
| 4.2 | Shipping | Describe how samples were stored and  shipped to the laboratory. | STORMS | Include length of time from collection to receipt  by the lab and if temperature control was used during shipping. | YES | Faecal sample  collection and storage |
| 4.3 | Storage | Describe how the laboratory stored samples,  including time between collection and storage and any preservation buffers or refrigeration used. | STORMS | State where each procedure or lot of samples was  done if not all in the same place. Include reagent/lot/catalogue #s for storage buffers. | YES | DNA sequencing and bioinformatics |
| 4.4 | DNA extraction | Provide DNA extraction method, including  kit and version if relevant. | MIxS:  nucleic  acid extraction | If any DNA quantification methods  were used prior to DNA amplification or at the pooling step of library preparation, state so here. | YES | DNA sequencing and bioinformatics |
| 4.5 | Human DNA  sequence depletion or microbial DNA enrichment | Describe whether human DNA sequence depletion or enrichment of microbial or viral DNA was performed. | STORMS |  | YES | DNA sequencing and bioinformatics |
| 4.6 | Primer selection | Provide primer selection and DNA amplification  methods as well as variable region sequenced  (if applicable). | MIxS: pcr  primers |  | YES | DNA sequencing and bioinformatics |
| 4.7 | Positive Controls | Describe any positive controls  (mock communities) if used. | STORMS | If used, should be deposited  under guidance provided in the 8.X items. | YES | DNA sequencing  and bioinformatics |
| 4.8 | Negative Controls | Describe any negative controls if used. | STORMS | If used, should be deposited under guidance provided in the  8.X items. | YES | DNA sequencing and bioinformatics |
| 4.9 | Contaminant  mitigation  and identification | Provide any laboratory or computational methods  used to control for or identify microbiome contamination from the environment, reagents, or laboratory. | STORMS | Includes filtering of reagents and other steps to minimize  contamination. It is relevant to state whether the specimens of interest have low microbial load, which makes contamination especially relevant. | NA |  |
| 4.10 | Replication | Describe any biological or technical replicates included  in the sequencing, including which steps were replicated between them. | STORMS | Replication may be biological (redundant biological  specimens) or technical (aliquots taken at different stages of analysis) and used in extraction, sequencing, preprocessing, and/or data analysis. | NA |  |
| 4.11 | Sequencing strategy | Major divisions of strategy, such as shotgun or  amplicon sequencing. | MIxS:  sequencing method | For amplicon sequencing  (for example, 16S variable region), state the region selected. State the model of sequencer used. | YES | DNA sequencing and bioinformatics |
| 4.12 | Sequencing methods | State whether experimental quantification was used  (QMP/cell count based, spike-in based) or whether relative abundance methods were applied. | STORMS | These include read length,  sequencing depth per sample (average and minimum), whether reads are paired, and other parameters. | YES | DNA sequencing and bioinformatics |
| 4.13 | Batch effects | Detail any blocking or randomization used in  study design to avoid confounding of batches with exposures or outcomes. Discuss any likely sources of batch effects, if known. | STORMS | Sources of batch effects include sample collection,  storage, library preparation, and sequencing and are commonly unavoidable in all but the smallest of studies. | YES | DNA sequencing and bioinformatics |
| 4.14 | Metatranscriptomics | Detail whether any mRNA enrichment was  performed and whether/how retrotranscription was performed prior to sequencing. Provide size range of isolated transcripts. Describe whether the sequencing library was stranded or not. Provide details on sequencing methods and platforms. | STORMS | Provide details on any internal standards which may have been  used as well as parameters and versions of any software or databases used. | NA |  |
| 4.15 | Metaproteomics | Detail which protease was used for digestion.  Provide details on proteomic methods and platforms (e.g. LC-MS/MS, instrument type, column type, mass range, resolution, scan speed, maximum injection time, isolation window, normalised collision energy, and resolution). | STORMS | Provide details on any internal standards which may have  been used as well as parameters and versions of any software or databases used. | NA |  |
| 4.16 | Metabolomics | Specify the analytic method used (such as nuclear  magnetic resonance spectroscopy or mass spectrometry). For mass spectrometry, detail which fractions were obtained (polar and/or non-polar) and how these were analyzed. Provide details on metabolomics methods and platforms (e.g. derivatization, instrument type, injection type, column type and instrument settings). | STORMS | Provide details on any internal standards which may have  been used as well as parameters and versions of any software or databases used. | NA |  |
| 5.0 | Data sources/  measurement | For each non-microbiome variable, including the health condition, intervention, or other variable of interest, state how it was defined, how it was measured or collected, and any transformations applied to the variable prior to analysis. | MIxS: host  disease  status | State any sources of potential bias in measurements,  for example multiple interviewers or measurement instruments, and whether these potential biases were assessed or accounted for in study design.  Use terms from a standardized ontology such as the  Experimental Factor Ontology (https://www.ebi.ac.uk/efo/) to describe variables of interest in a standardized format. | YES | Study population,  Other Characteristics |
| 6.0 | Research design  for causal inference | Discuss any potential for confounding by variables  that may influence both the outcome and exposure of interest. State any variables controlled for and the rationale for controlling for them. | STORMS | For causal inference, this item refers to describing the  assumptions that would be required to draw causal inferences from observational data. See Vujkovic-Cvijin, I., Sklar, J., Jiang, L. et al. Host variables confound gut microbiota studies of human disease. Nature 587, 448–454 (2020). https://doi.org/10.1038/s41586-020-2881-9 for more details on confounding in observational microbiome studies.  For example, hypothesized confounders may be controlled for by multivariable adjustment. Consider using a directed acyclic graph (DAG) to describe your causal model and justify any variables controlled for. DAGs can be made using [www.dagitty.net](http://www.dagitty.net/). | YES | Results, Table 1,  Adjustment for diet, Discussion |
| 6.1 | Selection bias | Discuss potential for selection or survival bias. | STORMS | Selection bias  can occur when some members of the target study population are more likely to be included in the study/final analytic sample than others. Some examples include survival bias (where part of the target study population is more likely to die before they can be studied), convenience sampling (where members of the target study population are not selected at random), and loss to follow-up (when probability of dropping out is related to one of the things being studied). | NA |  |
| 7.0 | Bioinformatic and  Statistical Methods | Describe any transformations to quantitative variables  used in analyses (e.g. use of percentages instead of counts, normalization, rarefaction, categorization). | STORMS | If a variable is analyzed using different transformations,  state rationale for the transformation and for each analyses which version of the variable is used.  In case of any complex or multistep  transformations, give enumerated instructions for reproducing those transformations. | YES | Statistical analysis |
| 7.1 | Quality Control | Describe any methods to identify or filter low quality  reads or samples. | MIxS:  sequence quality check | If samples  were excluded based on quality or read depth, list the criteria used, the number of samples excluded, and the final sample size after quality control. | YES | DNA sequencing and bioinformatics |
| 7.2 | Sequence analysis | Describe any taxonomic, functional profiling, or  other sequence analysis performed. | MIxS:  feature prediction; similarity search method |  | YES | DNA sequencing and bioinformatics |
| 7.3 | Statistical methods | Describe all statistical methods. | Modified  STROBE | Describe any statistical tests used, exploratory data  analysis performed, dimension reduction methods/unsupervised analysis, alpha/beta metrics, and/or methods for adjusting for measurement bias.  If multiple statistical methods are possible,  discuss why the methods used were selected.  If a multiple hypothesis testing correction method  was used, describe the type of correction used.  State which taxonomic levels are analyzed. | YES | Statistical analysis |
| 7.4 | Longitudinal  analysis | If the study is longitudinal, include a section that  explicitly states what analysis methods were used (if any) to account for grouping of measurements by individual or patterns over time. | STORMS |  | NA |  |
| 7.5 | Subgroup analysis | Describe any methods used to examine subgroups and interactions. | STROBE |  | NA |  |
| 7.6 | Missing data | Explain how missing data were addressed. | STROBE | "Missing data" refers to participant measurements  such as covariates, exposures, outcomes, or time points that should have been collected but were not, not to zeros in taxonomic abundance tables or data points not applicable to that observation. | YES | Statistical analyses,  Results |
| 7.7 | Sensitivity analyses | Describe any sensitivity analyses. | STROBE |  | YES | Statistical analysis |
| 7.8 | Findings | State criteria used to select findings for reporting. | STORMS | For example,  false discovery rate with total number of tests, effect size threshold, significance threshold, microbes of interest. | YES | Statistical analyses |
| 7.9 | Software | Cite all software (including read mapping software) and databases (including any used for taxonomic reference or annotating amplicons, if applicable) used. Include version numbers. | Modified  STREGA | Installed packages, add-ons or libraries should  be stated and cited in addition to the software used.  All parameters employed that differ from the default of that software/version should be provided.  This is in addition to,  not a replacement for, publishing of code as outlined in the section Reproducible Research. | YES | Statistical analyses |
| 8.0 | Reproducible  research | Make a statement about whether and how others  can reproduce the reported analysis. | STORMS | Any protected information that has been excluded  or provided under controlled access should be listed along with any relevant data access procedures. "On request from authors" is not sufficiently detailed; formal data access procedures and conditions should be defined.  If data are unavailable, state so clearly.  Consider using a specialized rubric for reproducible  research (such as:<https://mbio.asm.org/content/9/3/e00525-18.short)>.  Consider preregistering the study protocol (such as o[n osf.](http://osf.io/)io or<https://plos.org/open-science/preregistration/).> | YES | Data sharing statement |
| 8.1 | Raw data access | State where raw data may be accessed including demultiplexing information. | STORMS | Robust, long-term databases such as those hosted by  NCBI and EBI are preferred. If using a private repository, provide rationale. | YES | Data sharing statement |
| 8.2 | Processed  data access | State where processed data may be accessed. | STORMS | Unfiltered data should be provided.  Robust, long-term databases such as those hosted by  NCBI and EBI-EMBL are preferred. Repositories like zenodo (https://zenodo.org/) or publisso (https://www.publisso.de/en/working-for-you/doi-service/)  can be used to provide a DOI and long-term  storage for processed datasets, even those which cannot be published openly. | YES | Data sharing statement |
| 8.3 | Participant  data access | State where individual participant data such as  demographics and other covariates may be accessed, and how they can be matched to the microbiome data. | STORMS | If re-categorized, transformed, or otherwise derived variables  were used in the analysis, these variables or code for deriving them should be provided.  Examples of  how participant data can be matched to microbiome  data are: using the same set of anonymized identifiers, or using different anonymized identifiers but providing a map.  Provided data should be sufficient to independently replicate  the current analysis. | YES | Data sharing statement |
| 8.4 | Source code access | State where code may be accessed. | STORMS | If a standard or formalized workflow was employed,  reference it here. | YES | Supplementary |
| 8.5 | Full results | Provide full results of  all analyses, in computer-readable format, in supplementary materials. | STORMS | For example, any fold-changes, p-values, or FDR  values calculated, provided as a spreadsheet.  Use a machine-readable, plain-text format such as csv or tsv. | NO |  |
| **Results** | | | | | | |
| 9.0 | Descriptive data | Give characteristics of study participants  (e.g. dietary, demographic, clinical, social) and information on exposures and potential confounders. | STROBE | Typically reported in a table included in the paper or as  a supplementary table. Indicate number of participants with missing data for each variable of interest.  This includes environmental and lifestyle factors that  may affect the relationship between the microbiome and the condition of interest. Participant diet and medication use should be summarized, if known.  At minimum,  age and sex of all participants should be summarized. | YES | Results, Table 1 |
| 10.0 | Microbiome data | Report descriptive findings for microbiome  analyses with all applicable outcomes and covariates. | STORMS | This includes  measures of diversity as well as relative abundances. These descriptive findings should be reported both for the sample overall and for individual groups. | YES | Results |
| 10.1 | Taxonomy | Identify taxonomy using standardized taxon  classifications that are sufficient to uniquely identify taxa. | STORMS | If not using full  taxonomic hierarchy, make sure it is clear whether names stated are species, genera, family, etc.  Italicize genus/species pairs.  Consult journal guidelines or standardized references on taxonomic nomenclature. For instance,<https://wwwnc.cdc.gov/eid/page/scientific-nomenclature> | YES | Results |
| 10.2 | Differential  abundance | Report results of differential abundance analysis  by the variable of interest and (if applicable) by time, clearly indicating the direction of change and total number of taxa tested. | STORMS | If there are more than two groups, include omnibus  (multigroup) test results if applicable to the research question.  If applicable, reported effect sizes should include a measure of uncertainty such as the confidence interval. | YES | Differential abundance analyses |
| 10.3 | Other data types | Report other data analyzed--e.g. metabolic  function, functional potential, MAG assembly, and RNAseq. | STORMS |  | NA |  |
| 10.4 | Other  statistical analysis | Report any statistical data analysis not covered above. | STORMS | This could include subgroup analysis,  sensitivity analyses, and cluster analysis.  Visualizations should be  easily interpretable and colorblind-friendly. The caption and/or main text should provide a detailed description of visualizations for visually-impaired readers. | YES | Results |
| **Discussion** | | | | | | |
| 11.0 | Key results | Summarise key results with reference to  study objectives | STROBE |  |  | Discussion |
| 12.0 | Interpretation | Give a cautious overall interpretation of  results considering objectives, limitations, multiplicity of analyses, results from similar studies, and other relevant evidence. | STROBE | Define or clarify any subjective terms such as "dominant,"  "dysbiosis," and similar words used in interpretation of results.  When interpreting the findings, consider how the  interpretation of the findings may be summarized or quoted for the general public such as in press releases or news articles.  If causal language is used in the interpretation (such as  "alters," "affects," "results in," "causes," or "impacts"), assumptions made for causal inference should be explicitly stated as part of 6.0 and 13.0.  Distinguish between function potential (ie inferred from  metagenomics) and observed activity (ie metatranscriptomic, metabolomic, proteomic) if discussing microbial function. | YES | Discussion |
| 13.0 | Limitations | Discuss limitations of the study, taking into account  sources of potential bias or imprecision. | STROBE | Also consider limitations resulting from  the methods (especially novel methods), the study design, and the sample size. | YES | Limitations |
| 13.1 | Bias | Discuss any potential for bias to influence study  findings. | STORMS | May include sampling method, representativeness of study  participants, or potential confounding. | YES | Limitations |
| 13.2 | Generalizability | Discuss the generalisability (external validity)  of the study results | STROBE | To what populations or other settings do you expect the  conclusions to generalize? | YES | Introduction, Discussion |
| 14.0 | Ongoing/future  work | Describe potential future research or ongoing  research based on the study's findings. | STORMS |  | YES | Conclusions |
| **Other information** | | | | | | |
| 15.0 | Funding | Give the source of funding and the role of the funders  for the present study and, if applicable, for the original study on which the present article is based | STROBE |  | YES | Funding |
| 15.1 | Acknowledgements | Include acknowledgements of those  who contributed to the research but did not meet critera for authorship. | STORMS | For general guidelines on authorship,  see [http://www.icmje.org](http://www.icmje.org/) and<https://www.elsevier.com/authors/journal-authors/policies-and-ethics/credit-author-statement> | YES | Contributors |
| 15.2 | Conflicts of Interest | Include a conflicts of interest statement. | STORMS |  | YES | Competing interest |
| 16.0 | Supplements | Indicate where supplements may be  accessed and what materials they contain. | STORMS |  | YES | Supplements |
| 17.0 | Supplementary data | Provide supplementary data files of results  with for all taxa and all outcome variables analyzed. Indicate the taxonomic level of all taxa. | STORMS | Depending on the analysis performed, examples  of the supplemental results included could be mean relative abundance, differential abundance, raw p-value, multiple hypothesis testing-adjusted p-values, and standard error.  All discussed taxa should include the taxonomic  level (e.g. class, order, genus). | NO |  |

Supplemental table 2 S3: Diet score food groups and description

|  | |
| --- | --- |
| **Food group** | **Scoring/Description** |
| **Favorable food groups** | **Quartiles scored conversely: lowest consumption 0 points and highest consumption 3 points.** |
| Fiber-rich grain products | Bread, flour and flakes with fiber content ≥5g / 100g. Excludes snacks, biscuits, cereals, muesli, snack bars. |
| Fruits and berries | Fresh and frozen. Excludes juices, jams, dried or canned fruits and berries. |
| Vegetables | Fresh and frozen edible roots, corn, pulses and sprouts. Excludes potatoes, mushrooms, preserves, soups and soy products. |
| Fish | Fresh, smoked and canned fish, shrimp, crab and crayfish. Excludes fish eggs, mussels, squid and salt fish. |
| Nuts and seeds^1^ | Unflavored. Excludes salted, sugar coated or flavored nuts/seeds. |
| Low-fat, unsweetened dairy | Milk, fermented milk, quark and fermented curdled milk with ≤1%, cottage cheese and vegetable fat cheese ≤17%. Excludes creams and flavored dairy products. |
| Vegetable-oil based fats | Oils, margarines, flavored oils and salad dressings. Excludes mayonnaises and baking margarines. |
| **Unfavorable food groups** | **Quartiles scored inversely: highest consumption 0 points and lowest consumption 3 points.** |
| Red and processed meat | Porcine, bovine and ovine meat, sausages, pate and cold cuts. Excludes offal. |
| Sugar-sweetened beverages | Juices, soft drinks and energy drinks with sugar ≥0.6g / 100ml. Excludes alcohol, natural fruit juices, protein shakes and sports drinks. |
| Salty snacks^2^ | Potato chips, salted nuts, popcorn, cheese-flavored corn snacks. |
| Desserts | Puddings, ice cream, chocolate, cocoa drink powder, sugar. |
| ^1^Low consumption of nuts and seeds; any consumption scored 3, no consumption scored 0  ^2^Low consumption of salty snacks; any consumption scored 0, no consumption scored 3 | |

Supplemental table 3 S4: Differential abundance analysis results. baseMean refers to mean abundance, log2FC to log2 of fold-change between the LTPA groups, lfcSE to standard error of log2 of fold-change, adj.p refers to the adjusted p-value.

|  | **baseMean** | **log2FC** | **lfcSE** | **adj.p** |
| --- | --- | --- | --- | --- |
| **Haemophilus** | 175.9 | -1.4 | 0.32 | < 0.001 |
| **Family: Porphyromonadaceae** | 10.6 | -5.8 | 1.40 | 0.002 |
| **Lactobacillus** | 23.9 | -2.3 | 0.64 | 0.01 |
| **Paraprevotella** | 10265.7 | -2.2 | 0.63 | 0.01 |
| **Veillonella** | 442.3 | -0.9 | 0.27 | 0.01 |
| **Prevotella** | 146169.3 | -1.6 | 0.47 | 0.02 |
| **Papillibacter** | 29.5 | 1.7 | 0.56 | 0.03 |
| **Salmonella** | 23.8 | -2.4 | 0.81 | 0.03 |
| **Streptococcus** | 283 | -0.7 | 0.23 | 0.03 |
| **Clostridium XlVa** | 4652.4 | -0.3 | 0.10 | 0.04 |
| **Phylum: Proteobacteria** | 139.6 | -2.3 | 0.81 | 0.04 |
| **Romboutsia** | 97 | -0.9 | 0.30 | 0.04 |
| **Coprococcus** | 1448.6 | -0.7 | 0.27 | 0.04 |
| **Anaerostipes** | 291.3 | -0.6 | 0.22 | 0.04 |
| **Subdoligranulum** | 74 | 0.7 | 0.244 | 0.04 |
| **Barnesiella** | 11651.1 | -1.1 | 0.40 | 0.04 |
| **Fusobacterium** | 2.4 | -2.8 | 1.07 | 0.05 |
| **Streptophyta** | 38.8 | -1.4 | 0.5132 | 0.0482 |

*Supplemental Information 1: Results after exclusion of those treated with antibiotics or missing antibiotic information.*

We found no significant differences in alpha diversity assessed as Shannon index and observed richness between the active and inactive groups (Wilcoxon p=0.11 and p=0.14). The beta dispersion analysis between active and inactive groups was significant (p=0.017), which indicates that the groups are different regarding groups internal homogeneousness. In the PERMANOVA analysis the observed community compositions differed between the groups regarding Bray-Curtis dissimilarity (R^2^=0.008, p=0.012) on ASV-level.

After the participants treated with antibiotics or missing antibiotic information were excluded the *DESeq2* analysis identified 18 *genera* that differed between the active and inactive LTPA groups (Supplemental Figure 2 S5)*.* These *genera* remained mostly the same. The inactive group had a higher abundance of *Subdoligranulum* (adj.p = 0.033). The active group had higher abundance of *Paraprevotella* (adj.p = 0.001), *Porphyromonadaceae* (adj.p = 0.001), *Haemophilus* (adj.p = 0.001), *Prevotella* (adj.p = 0.002), *Megasphaera* (adj.p = 0.003), *Lactobacillus* (adj.p = 0.004), *Streptophyta* (adj.p = 0.033), *Coprococcus* (adj.p = 0.033), *Streptococcus* (adj.p = 0.033), *Clostridium XlVa* (adj.p = 0.033), *Romboutsia* (adj.p = 0.035), unknown *Proteobacteria* (adj.p = 0.047), *Veillonella* (adj.p = 0.047), *Anaerostipes* (adj.p = 0.047), unknown *Lachnospiraceae* (adj.p = 0.047), *Fusobacterium* (adj.p = 0.048), and *Salmonella (adj.p = 0.05).*

Lastly, we repeated the PERMANOVA and DA analyses with diet score added to models without those treated with antibiotics (N=250, active=178, inactive=72). The PERMANOVA analysis remained significant between the LTPA groups and community composition regarding Bray-Curtis dissimilarity (R^2^=0.007, p=0.023), even with diet on the models. The strongest associations were found for the genera *Prevotella, Paraprevotella, Ruminococcus, Bacteroides, Roseburia*, and *Faecalibacterium*. The elevated abundance of certain *Prevotella* and *Paraprevotella* species in the active group and genus *Bacteroides* in the inactive group drive the difference in beta diversity. The DA analysis identified 7 genera that differed between the active and inactive LTPA groups (Supplemental Figure 3 S6). The inactive group had a higher abundance of *Bacteroides* (adj.p = 0.002), *Hungatella* (adj.p = 0.048), and *Blautia* (adj.p = 0.048). The active group had a higher abundance of four genera: unknown Firmicutes (adj.p = 0.001), *Barnesiella* (adj.p = 0.001), *Lachnospiraceae* (adj.p = 0.007), and unknown Bacteria (adj.p = 0.008).


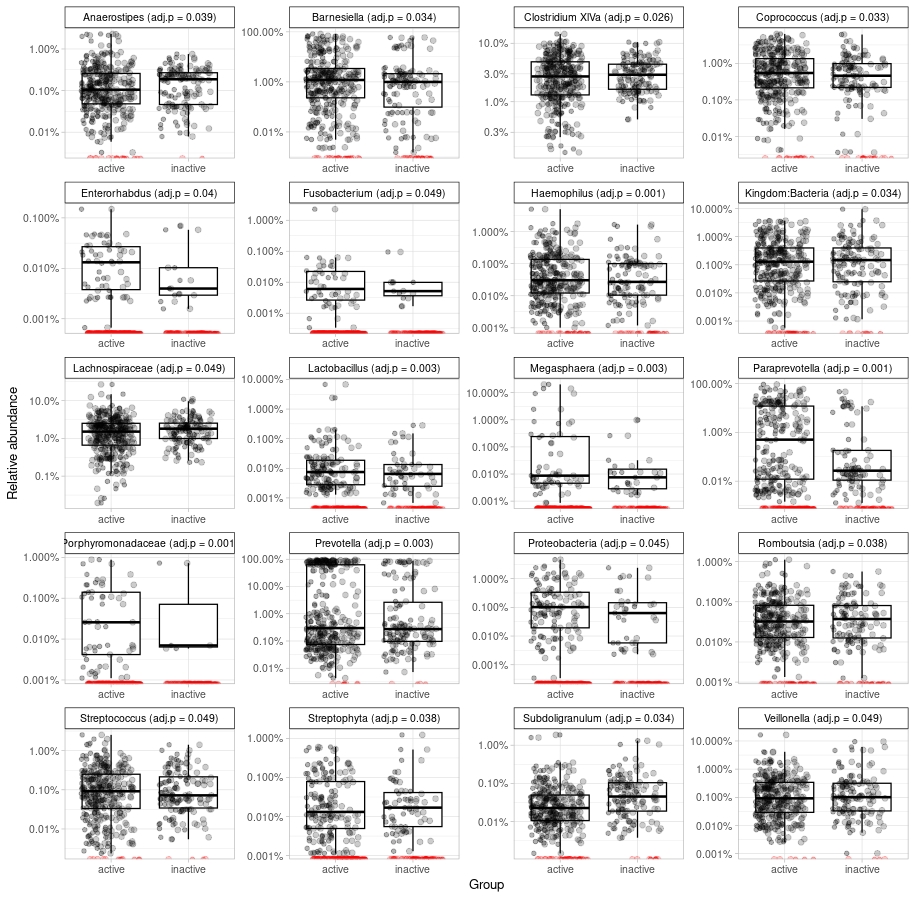


Supplemental figure 2 S5: Relative abundance of those genera that were identified as differentially abundant in active and inactive groups before adding diet to model (black dots: abundance > 0, red dots: abundance = 0). The adjusted p-values (adj.p) are obtained from the DESeq2 analysis. The antibiotic treated excluded from the analysis (N=269)


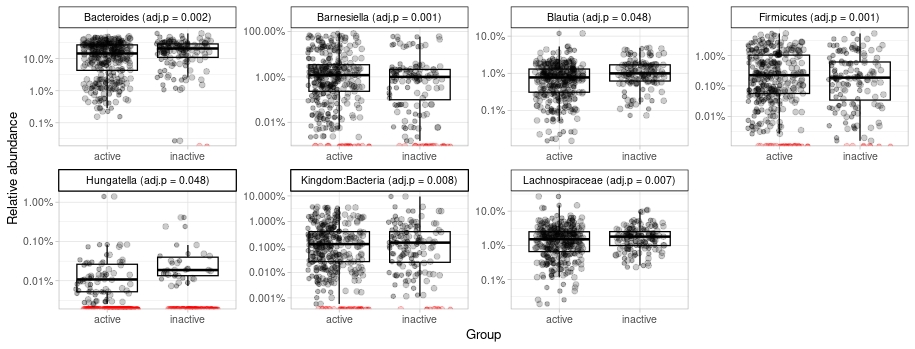


Supplemental figure 3 S6: Relative abundance of those genera that were identified as differentially abundant in active and inactive groups after adding diet to model (black dots: abundance > 0, red dots: abundance = 0). The adjusted p-values (adj.p) are obtained from the DESeq2 analysis. The antibiotic treated are excluded from the analysis (N=250 remaining in the analysis).

*Supplementary methods: R script*

library(haven)
library(dplyr)

library(mia)

library(vegan)

library(scater)

library(Biostrings)

library(ggplot2)

library(readxl)

library(table1)

library(tibble)

library(BiocManager)

library(boot)

library(scater)

library(knitr)

library(kableExtra)

library(DESeq2)

library(patchwork)

library(tidySummarizedExperiment)

library(cowplot)

library(tidyverse)

library(ggsignif)

setwd("~/STRIP1/Data")

**# Tax_table###**

tax_table <- read.csv("ASVs.csv", sep = ";", row.names = 1)

tax_table <- DataFrame(tax_table)

**# count-table**

asv_table <- read.csv("ASVs_full_korj.csv", sep = ";", row.names = 1)
assay_full <- t(as.matrix(asv_table))

**#Used in filtering subject data**

in_assay <- colnames(assay_full)

**#Data import####**

strip_data1 <- haven::read_sas("strip_26v.sas7bdat")

strip_data1 <- strip_data1 %>%

filter(IDF %in% in_assay)

df <- strip_data1

df <- df %>%

add_column(Group = case_when(.$met <= 5 ~ 'inactive', .$met >= 16 ~ 'active'), .after = "met")

strip_data1 <- df

diet <- haven::read_sas("dietscore_26v.sas7bdat")

diet <- diet%>%

filter(IDF%in% in_assay)

strip_metadata_uusi <- read_sas("strip_metadata_uusi.sas7bdat")

#tässä antibiootit, Batchit

strip_metadata_uusi <- strip_metadata_uusi[, c("_SampleID","Batch","Antibioottikuuri")]#poistetaan
 #Sukupuoli, interventioryhmä ym. muut tuplat
 strip_metadata_uusi <- strip_metadata_uusi %>%
 filter(`_SampleID` %in% in_assay)
 #Kaikki metatiedot uuteen dataframeen
 tab1 = strip_data1
 tab2 = strip_metadata_uusi
 tab3 = diet
 data_yhd <-merge(x=tab1,y=tab2,by.x="IDF",by.y = "_SampleID",all= T)
 diet_yhd <-merge(x=tab1,y=tab3,by.x="IDF",by.y = "IDF",all= T)
 tab4 = diet_yhd
 diet_yhd <-merge(x=tab4,y=tab2,by.x="IDF",by.y = "_SampleID",all= T)
 #factors
 diet_yhd$SP <- factor(diet_yhd$SP,
 levels = c(1, 2),
 labels = c("female", "male"))
 diet_yhd$diabetes26v <- factor(diet_yhd$diabetes26v,
 levels = c(0, 1),
 labels = c("no", "yes"))
 diet_yhd$regular_smoke <- factor(diet_yhd$regular_smoke,
 levels = c(0, 1),
 labels = c("no", "yes"))
 diet_yhd$RASKAU <- factor(diet_yhd$RASKAU,
 levels = c(0, 1, 2),
 labels = c("0", "1", "2"))
 diet_yhd$IMETYS <- factor(diet_yhd$IMETYS,
 levels = c(0, 1, 2),
 labels = c("0", "1", "2"))
 diet_yhd$RRLAA <- factor(diet_yhd$RRLAA,
 levels = c(0, 1),
 labels = c("0", "1"))
 diet_yhd$KOLLAA <- factor(diet_yhd$KOLLAA,
 levels = c(0, 1),
 labels = c("0", "1"))
 diet_yhd$osr2 <- factor(diet_yhd$osr2,
 levels = c(1, 2),
 labels = c("control", "intervention"))
 diet_yhd$Antibioottikuuri <- factor(diet_yhd$Antibioottikuuri,
 levels = c(0, 1),
 labels = c("no", "yes"))
 diet_yhd$Group <- factor(diet_yhd$Group,
 levels = c( "active", "inactive"),
 labels = c("active", "inactive"))
 to_coldata <- diet_yhd

**#####BATCH in Group, Chi square**
 # Perform chi-square test of independence

 chi_square_result <- chisq.test(diet_yhd$Batch, diet_yhd$Group)
  **# Create TSE for microbiome data**
 assays <- SimpleList(counts = assay_full)
 # Create TreeSE from full data

tse <- TreeSummarizedExperiment(assays = assays, rowData = tax_table, colData = to_coldata)

TSE <- tse

**#Read count######**

tsecounts <- perCellQCMetrics(tse) summary(tsecounts$sum)

TSE<- transformSamples(TSE, method = "relabundance")

altExp(TSE, "Genus") <- agglomerateByRank(TSE, rank="Genus")

TSE_Groups <- TSE[ , TSE$Group %in% c("active", "inactive")]

tse <- TSE_Groups

**#Alpha diversity###**

#Shannon

tse <- mia::estimateDiversity(tse, assay.type = "counts", index = "shannon", name = "shannon") head(tse$shannon)

spl <- split(tse$shannon, tse$Group)

**#Kolmogorov-Smirnov test**

pv <- ks.test(spl$active, spl$inactive)$p.value pv mean(spl$active)#3.171984 mean(spl$inactive)#3.365136 wilcoxon_shannon <- wilcox.test(tse$shannon ~ Group, data = colData(tse)) df <- as.data.frame(colData(tse)[tse$Group %in% c("active", "inactive"),]) df$Group <- factor(df$Group)

comb <- split(t(combn(levels(df$Group), 2)), seq(nrow(t(combn(levels(df$Group), 2)))))

p.shannon <- ggplot(df, aes(x = Group, y = shannon, fill = Group)) +

geom_boxplot(outlier.shape = NA, alpha = 0.5) + geom_jitter(width = 0.2) + #geom_signif(comparisons = comb, map_signif_level = FALSE) + theme(text = element_text(size = 10))+ theme_light()+ scale_fill_manual(values = c("active" = "#56B4E9", "inactive" = "#CC79A7"))

print(p.shannon)

**#Richness ###**

tse <- TSE_Groups

tse <- mia::estimateRichness(tse, assay.type = "counts", index = "observed", name = "observed") head(tse$observed)

spl <- split(tse$observed, tse$Group)

**#Kolmogorov-Smirnov test**

pv <- ks.test(spl$active, spl$inactive)$p.value

pv

mean(spl$active)#244.73

mean(spl$inactive)#263,16

wilcoxon_observed <- wilcox.test(tse$observed ~ Group, data = colData(tse))

df <- as.data.frame(colData(tse)[tse$Group %in% c("active", "inactive"),])

df$Group <- factor(df$Group)

comb <- split(t(combn(levels(df$Group), 2)), seq(nrow(t(combn(levels(df$Group), 2)))))

p.richness <- ggplot(df, aes(x = Group, y = observed, fill = Group)) + geom_boxplot(outlier.shape = NA, alpha = 0.5) + geom_jitter(width = 0.2) +

geom_signif(comparisons = comb, map_signif_level = FALSE) +

theme_light()+ theme(text = element_text(size = 10)) + scale_fill_manual(values = c("active" = "#56B4E9", "inactive" = "#CC79A7"))

print(p.richness)

#cairo_ps(file = "Figure1", onefile = FALSE, fallback_resolution = 600)

#plot_grid(p.shannon, p.richness, labels = "AUTO", label_size = 0)

dev.off()

**#Beta diversity: Bray####**

tse <- TSE_Groups #Checking homogeneity

anova( betadisper(vegdist(t(assay(tse, "counts"))), colData(tse)$Group)) #Which group has the girher dispersion

dis <- vegan::vegdist(t(assays(tse)$relabundance), method = "bray")

b <- vegan::betadisper(dis, colData(tse)$Group) anova(b)

**#PERMANOVA####**

set.seed(2435) permanova <- adonis2(t(assay(tse, "relabundance")) ~ Group, by = "margin", data = colData(tse), method = "bray", permutations = 999)

Permanova

**#PCOA_Plot##**

tse <- TSE_Groups

tse <- runMDS(tse, FUN = vegan::vegdist, method = "bray", name = "PCoA_BC", exprs_values = "relabundance")

p <- plotReducedDim(tse, "PCoA_BC", colour_by = "Group")

e <- attr(reducedDim(tse, "PCoA_BC"), "eig") rel_eig <- e / sum(e[e > 0]) p <- p + labs(x = paste("PCoA 1 (", round(100 * rel_eig[[1]], 1), "%", ")", sep = ""), y = paste("PCoA 2 (", round(100 * rel_eig[[2]], 1), "%", ")", sep = ""))

p <- p + scale_color_manual(values = c("active" = "#56B4E9", "inactive" = "#CC79A7"), name = "Group") + theme(text = element_text(size = 12))

ggsave("Figure2.eps", plot=p, device = cairo_ps)

**#PCOA-plot Dominant abundance**

# Agglomerate to genus level

tse_genus <- agglomerateByRank(tse, rank = "Genus")

# Convert to relative abundances

tse_genus <- transformAssay(

tse, method = "relabundance", assay.type = "counts")

# Add info on dominant genus per sample

tse_genus <- addDominant(

tse_genus, assay.type = "relabundance", name = "dominant_taxa")

# Overview

summarizeDominance(

tse_genus, rank = "Genus", digits = 3, name = "dominant_taxa")

## # A tibble: 17 × 3

## dominant_taxa n rel_freq

## <chr> <int> <dbl>

## 1 Bacteroides 5 0.192

## 2 Crenothrix 3 0.115

## 3 Faecalibacterium 2 0.077

## 4 Prochlorococcus 2 0.077

## 5 Streptococcus 2 0.077

## 6 CandidatusNitrososphaera 1 0.038

## # ℹ 11 more rows

tse_genus <- runMDS(

tse_genus,

FUN = getDissimilarity,

name = "PCoA_BC",

method = "bray",

assay.type = "relabundance")

# Getting the top taxa

top_taxa <- getTop(tse_genus, top = 6, assay.type = "relabundance")

# Naming all the rest of non top-taxa as "Other"

most_abundant <- lapply(

colData(tse_genus)$dominant_taxa, function(x){

if (x %in% top_taxa) {x} else {"Other"}

})

# Storing the previous results as a new column within colData

colData(tse_genus)$most_abundant <- as.character(most_abundant)

# Calculating percentage of the most abundant

most_abundant_freq <- table(as.character(most_abundant))

most_abundant_percent <- round(

most_abundant_freq / sum(most_abundant_freq) * 100,

1)

# Retrieving the explained variance

e <- attr(reducedDim(tse_genus, "PCoA_BC"), "eig")

var_explained <- e / sum(e[e > 0]) * 100

# Define colors for visualization

my_colors <- c(

"black", "blue", "lightblue", "darkgray", "magenta", "darkgreen", "red")

# Visualization

p2 <- plotReducedDim(tse_genus, "PCoA_BC", colour_by = "most_abundant") +

scale_colour_manual(

values = my_colors,

labels = paste0(

names(most_abundant_percent), "(", most_abundant_percent, "%)")) +

labs(

x = paste("PC 1 (", round(var_explained[1], 1), "%)"),

y = paste("PC 2 (", round(var_explained[2], 1), "%)"),

color = "")

p2

**#PLOTS P and P2 together**

> p + p2 +

+ plot_annotation(tag_levels = 'A') # Labels the plots as A and B

**#top taxa figure on GENUS LEVEL####**

altExp(tse,"Genus") <- subsetByPrevalentFeatures(altExp(tse,"Genus"), detection = 0, prevalence = 0.1)

tse <- altExp(tse,"Genus") set.seed(2435)

permanova3 <- adonis(t(assay(tse, "relabundance")) ~ Group, data = colData(tse), method = "bray", permutations = 999) #tämä 999

coef <- coefficients(permanova3)["Group1",]

coef <- as.data.frame(coef) head(coef)

#Plot

top.coef <- head( coef[rev(order(abs(coef$coef))), , drop = FALSE], 6) #20 #COEFF PLOT

top.coef <- top.coef %>%

rownames_to_column("taxa") %>%

mutate(taxa = str_replace(taxa, "Genus:", "")) %>%

arrange(coef) %>%

mutate(taxa = fct_reorder(taxa, coef))

coefp <- ggplot(top.coef, aes(coef, taxa)) + geom_bar(stat="identity") + labs(x="Coefficient", title="Coefficient", y= "Genus") + theme_bw()

ggsave("Figure3.eps", plot=coefp, device = cairo_ps)

**##DA on GENUS level###**

tse <- TSE_Groups altExp(tse,"Genus") <- subsetByPrevalentFeatures(altExp(tse,"Genus"), detection = 0, prevalence = 0.1)

tse <- altExp(tse,"Genus") #deseq
ds2 <- DESeqDataSet(tse, ~Group)

dds <- DESeq(ds2)

res <- results(dds)

df <- as.data.frame(res)

df$taxon <- rownames(df) rowData(tse)[, c("Genus","Family")]

df <- df %>% arrange(log2FoldChange, padj) df <- df %>% arrange(padj) df <- filter(df, padj < .05) df <- rownames_to_column(df, "rowname") knitr::kable(head(df)) %>% kableExtra::kable_styling("striped") %>% kableExtra::scroll_box(width = "100%") df %>% write_tsv("./deseq.txt") res_G <- read_tsv("./deseq.txt")

tse <- TSE_Groups
altExp(tse,"Genus") <- subsetByPrevalentFeatures(altExp(tse,"Genus"), detection = 0, prevalence = 0.1)
tse <- altExp(tse,"Genus")
tse <- transformCounts(tse,assay.type = "counts", method = "relabundance")
tse <- transformCounts(tse,assay.type = "relabundance", method = "rclr")

d <- as_tibble(t(assay(tse, "relabundance"))) %>%

mutate(Group = as.character(colData( tse)[ , c("Group")]), IDF = as.character(colData( tse)[ , c("IDF")])) %>%
dplyr::select(res_G$rowname, Group, IDF)

deseq_genus_plot <- d %>%

pivot_longer(cols = res_G$rowname, names_to = "rowname",

values_to = "abundance") %>%

# mutate(rowname = gsub("Genus:", "", rowname)) %>%

dplyr::left_join(res_G, by = "rowname") %>%

ggplot(., aes(Group, abundance)) +

#color = abundance == 0))

geom_boxplot(outlier.shape = NA) + geom_jitter(alpha = 0.3, size = 2, height = 0, #No jitter in vertical direction! width = .3) +

#scale_y_log10() scale_y_log10(label = scales::percent) + #scale_color_manual(values = c('back', 'red')) +

geom_point(alpha = 0.3, position = position_jitterdodge()) +

theme_light() +

theme(legend.position = "none") +

theme(legend.position = "none") +

ylab("Relative abundance") + facet_wrap( ~ rowname, scales = "free",ncol=4) #+facet_grid(~ DAMethod, scales = "free")

ggsave("Figure4.eps", plot=deseq_genus_plot, width =10, height = 8, units = "in" , device = cairo_ps)

**#Adjustment for the diet score######**

tse <- TSE_Groups

tse <- tse[, !is.na(tse$dietscore_E_lapsi)]

**#PERMANOVA#######**

set.seed(2435)

permanovaCO <- adonis2(t(assay(tse, "relabundance")) ~ Group +

dietscore_E_lapsi, by = "margin", data = colData(tse), method = "bray", permutations = 999) #tämä 999

permanovaCO

**#DESQ ######**

tse <- TSE_Groups ^

altExp(tse,"Genus") <- subsetByPrevalentFeatures(altExp(tse,"Genus"), detection = 0, prevalence = 0.1)

tse <- altExp(tse,"Genus") #deseq

ds2 <- DESeqDataSet(tse, ~Group + dietscore_E_lapsi)

dds <- DESeq(ds2)

res <- results(dds)**s**

df <- as.data.frame(res)

df$taxon <- rownames(df)

rowData(tse)[, c("Genus","Family")]

df <- df %>%

arrange(log2FoldChange, padj)

df <- df %>%

arrange(padj)

df <- filter(df, padj < .05)

df <- rownames_to_column(df, "rowname")

knitr::kable(head(df)) %>% kableExtra::kable_styling("striped") %>% kableExtra::scroll_box(width = "100%") df %>% write_tsv("./deseqco.txt") res<- read_tsv("./deseqco.txt")

**#object**

tse <- TSE_Groups altExp(tse,"Genus") <- subsetByPrevalentFeatures(altExp(tse,"Genus"), detection = 0, prevalence = 0.1) tse <- altExp(tse,"Genus") tse <- transformCounts(tse,assay.type = "counts", method = "relabundance") tse <- transformCounts(tse,assay.type = "relabundance", method = "rclr")

d <- as_tibble(t(assay(tse, "relabundance"))) %>% mutate(Group = as.character(colData( tse)[ , c("Group")]), IDF = as.character(colData( tse)[ , c("IDF")])) %>% dplyr::select(res$rowname, Group, IDF)

**#Black&ampwhite plot,**

deseq_co <- d %>%

pivot_longer(cols = res$rowname, names_to = "rowname", values_to = "abundance")%>% # tässä pitkäksi

mutate(rowname = gsub("Genus:", "", rowname)) %>%

filter(abundance != 0) %>%

dplyr::left_join(res, by = "rowname") %>%

ggplot(., aes(Group, abundance)) +

color = abundance == 0)) +

geom_boxplot(outlier.shape = NA) +

geom_jitter(alpha = .2, size = 2, height = 0,

#No jitter in vertical direction! width = .3) +

scale_y_continuous(labels = function(x) format(x, scientific = FALSE))+

scale_y_log10() +

geom_point(alpha = 0.3, position = position_jitterdodge()) +

theme_light() +

theme(legend.position = "none") +

theme(legend.position = "none") +

ylab("Relative abundance") +

facet_wrap( ~ rowname, scales = "free",ncol=4) #+

facet_grid(~ DAMethod, scales = "free")

deseq_co

**#Dispersion ######**

tse <- TSE_Groups

#Which group has the girher dispersion

dis <- vegan::vegdist(t(assays(tse)$relabundance), method = "bray")

b <- vegan::betadisper(dis, colData(tse)$Group) anova(b)

p <- cbind(distance = as.numeric(b$distances),

dietscore_E_lapsi = as.character

(colData(tse)$dietscore_E_lapsi) ) %>%

as_tibble() %>%

mutate(distance = as.numeric(distance)) %>%

ggplot(aes(dietscore_E_lapsi, distance, color = dietscore_E_lapsi)) + geom_boxplot(outlier.shape = NA) +

geom_jitter(alpha = 0.3) +

theme_light()

print(p)

**#Sensitivity ANTIBIOTICS####**

#We run the analysis excluding those treated with antibiotics or missing data with the similar pipeline as above.

tse <- tse[ , tse$Antibioottikuuri %in% c("no")]

**#TABLE 1####**

df <- as.data.frame(colData(TSE_Groups))

#factors

diet_yhd$SP <- factor(diet_yhd$SP,

levels = c(1, 2),

labels = c("female", "male"))

diet_yhd$diabetes26v <- factor(diet_yhd$diabetes26v,

levels = c(0, 1),

labels = c("no", "yes"))

diet_yhd$regular_smoke <- factor(diet_yhd$regular_smoke,

levels = c(0, 1),

labels = c("no", "yes"))

diet_yhd$RASKAU <- factor(diet_yhd$RASKAU,

levels = c(0, 1, 2),

labels = c("0", "1", "2"))

diet_yhd$IMETYS <- factor(diet_yhd$IMETYS,

levels = c(0, 1, 2),

labels = c("0", "1", "2"))

diet_yhd$RRLAA <- factor(diet_yhd$RRLAA,

levels = c(0, 1),

labels = c("0", "1"))

diet_yhd$KOLLAA <- factor(diet_yhd$KOLLAA,

levels = c(0, 1),

labels = c("0", "1"))

diet_yhd$osr2 <- factor(diet_yhd$osr2,

levels = c(1, 2),

labels = c("control", "intervention"))

diet_yhd$Antibioottikuuri <- factor(diet_yhd$Antibioottikuuri,

levels = c(0, 1),

labels = c("no", "yes"))

diet_yhd$Group <- factor(diet_yhd$Group,

levels = c( "active", "inactive"),

labels = c("active", "inactive"))

diet_yhd$aljuop <- factor(diet_yhd$aljuop,

levels = c(1, 2, 3, 4, 5, 6),

labels = c("2+x/wk","1x/wk", "2-3x/mth", "1x/mth", "2-6x/y", "Less"))

diet_yhd$tyo_rasitus <- factor(diet_yhd$tyo_rasitus,

levels = c(1, 2, 3, 4 ),

labels = c("1=primarely desk work","2= walk a fair amount at work", "3= walk, lift things, take the stairs or go uphill a lot", "4= physically strenuous job"))

diet_yhd$toimi <- factor(diet_yhd$toimi,

levels = c(1, 2, 3, 4, 5, 6, 7),

labels = c("1=Working full-time" , "2=Working, but studying as well" , "3=Studying full-time" , "4=Unemployed or temporarely laid off" , "5=Disability support pension" , "6=Stay-at-home mom or dad" , "7=Other"))

diet_yhd$reslaa <-factor(diet_yhd$reslaa,

levels = c(0, 1),

labels = c("no", "yes"))

df <- diet_yhd

diet_yhd <- diet_yhd%>%

filter(IDF%in% in_assay)

colnames(df)

df <- df %>%

dplyr::select('IDF', 'R1', 'SP', 'diabetes26v', 'PS91950_dessert','met', 'reslaa', 'aljuop', 'toimi', 'tyo_rasitus', 'Group', 'regular_smoke', 'bmi', 'syst',

'diast', 'waist', 'VO2mlkg4min', 'osr2', 'PS91800_allgrains', 'PS91810_fruitberries', 'PS91820_vegetables', 'PS91830_fish', 'PS91840_nut', 'PS91860_vegfat', 'PS91850_lfmilk', 'PS91900_redmeat', 'PS91920_beverages', 'PS91930_snacks', 'dietscore_E_lapsi', 'Batch', 'Antibioottikuuri' )

df <- subset(df, Group %in% c("active", "inactive"))

##NORMALITY OF NUMERICAL VARIABLES#####

#Histograms

library(reshape2)

# Convert df to long

df_long <- reshape2::melt(df) #

# Create histograms for each variable

ggplot(df_long, aes(x = value)) +

geom_histogram(bins = 30, fill = "blue", color = "black", alpha = 0.7) +

facet_wrap(~variable, scales = "free") + # Facet by variable name

theme_minimal() +

labs(title = "Histograms of All Variables", x = "Value", y = "Frequency")

label(df$R1) <- "Energy intake"

label(df$SP) <- "Sex"

label(df$diabetes26v) <- "Diabetes"

label(df$met) <- "Met/h/wk"

label(df$regular_smoke) <- "Smoking status"

label(df$bmi) <- "Bmi"

label(df$waist) <- "Waist (cm)"

label(df$osr2) <- "Strip study group"

label(df$PS91800_allgrains) <- "All grains g/d"

label(df$PS91810_fruitberries) <- "Fruit &amp Berries g/d"

label(df$PS91820_vegetables) <- "Vegetables g/d"

label(df$PS91830_fish) <- "Fish g/d"

label(df$PS91860_vegfat) <- "Vegetable fat g/d"

label(df$PS91850_lfmilk) <- "Milk ml/d"

label(df$PS91900_redmeat) <-"Red and processed meat g/d"

label(df$dietscore_lapsi) <- "Dietscore_child"

label(df$PS91920_beverages) <- "Sugar sweetened beverages"

label(df$PS91930_snacks ) <- "Salty snacks"

label(df$Antibioottikuuri) <- "Antibiotics"

label(df$dietscore_E_lapsi) <- "Diet score"

label(df$PS91950_dessert) <- "Desserts"

label(df$aljuop) <- "Heavy drinking"

label(df$reslaa) <- "Prescription drug"

label(df$toimi) <- "Current primary activity"

label(df$tyo_rasitus) <- "Occupational physical workload"

pvalue <- function(x, ...) {

# Construct vectors of data y, and groups (strata) g

y <- unlist(x)

g <- factor(rep(1:length(x), times=sapply(x, length)))

if (is.numeric(y)) {

# For numeric variables, perform a standard 2-sample t-test

p <- t.test(y ~ g)$p.value

} else {

# For categorical variables, perform a chi-squared test of independence

p <- chisq.test(table(y, g))$p.value

}

# Format the p-value, using an HTML entity for the less-than sign.

# The initial empty string places the output on the line below the variable label.

c("", sub("<", "&lt;", format.pval(p, digits=3, eps=0.001)))

}

table1::table1

(~ SP + osr2 + bmi + waist + diabetes26v + reslaa + Antibioottikuuri + VO2mlkg4min + aljuop + regular_smoke + toimi + tyo_rasitus + R1 + PS91950_dessert + PS91800_allgrains + PS91810_fruitberries + PS91820_vegetables + PS91830_fish + PS91840_nut + PS91860_vegfat + PS91850_lfmilk + PS91900_redmeat +PS91920_beverages + PS91930_snacks + dietscore_E_lapsi | Group, data=df, overall=F, extra.col=list(`P-value`=pvalue))

##IQR for non-normally distributed continuous variables

#Select non-normal based on histogram

df <- df %>%

dplyr::select('Group','PS91950_dessert', 'PS91810_fruitberries', 'PS91830_fish', 'PS91840_nut', 'PS91850_lfmilk', 'PS91900_redmeat', 'PS91920_beverages', 'PS91930_snacks')

#divide based on LTPA-group

dfac <- df %>%

filter(Group %in% c("active"))

dfin <- df %>%

filter(Group %in% c("inactive"))

#Calculate IGR values for each column

sapply(dfac,IQR,na.rm=TRUE)

sapply(dfin,IQR,na.rm=TRUE)

#Apply values to non-normally distributed variables based on Histograms
